# Genetic pleiotropy between age-related macular degeneration and 16 complex diseases and traits

**DOI:** 10.1186/s13073-017-0418-0

**Published:** 2017-03-27

**Authors:** Felix Grassmann, Christina Kiel, Martina E. Zimmermann, Mathias Gorski, Veronika Grassmann, Klaus Stark, International AMD Genomics Consortium (IAMDGC), Lars G. Fritsche, Wilmar Igl, Jessica N. Cooke Bailey, Felix Grassmann, Sebanti Sengupta, Jennifer L. Bragg-Gresham, Kathryn P. Burdon, Scott J. Hebbring, Cindy Wen, Mathias Gorski, Ivana K. Kim, David Cho, Donald Zack, Eric Souied, Hendrik P. N. Scholl, Elisa Bala, Kristine E. Lee, David J. Hunter, Rebecca J. Sardell, Paul Mitchell, Joanna E. Merriam, Valentina Cipriani, Joshua D. Hoffman, Tina Schick, Yara T. E. Lechanteur, Robyn H. Guymer, Matthew P. Johnson, Yingda Jiang, Chloe M. Stanton, Gabriëlle H. S. Buitendijk, Xiaowei Zhan, Alan M. Kwong, Alexis Boleda, Matthew Brooks, Linn Gieser, Rinki Ratnapriya, Kari E. Branham, Johanna R. Foerster, John R. Heckenlively, Mohammad I. Othman, Brendan J. Vote, Helena Hai Liang, Emmanuelle Souzeau, Ian L. McAllister, Timothy Isaacs, Janette Hall, Stewart Lake, David A. Mackey, Ian J. Constable, Jamie E. Craig, Terrie E. Kitchner, Zhenglin Yang, Zhiguang Su, Hongrong Luo, Daniel Chen, Hong Ouyang, Ken Flagg, Danni Lin, Guanping Mao, Henry Ferreyra, Klaus Stark, Claudia N. von Strachwitz, Armin Wolf, Caroline Brandl, Guenther Rudolph, Matthias Olden, Margaux A. Morrison, Denise J. Morgan, Matthew Schu, Jeeyun Ahn, Giuliana Silvestri, Evangelia E. Tsironi, Kyu Hyung Park, Lindsay A. Farrer, Anton Orlin, Alexander Brucker, Mingyao Li, Christine A. Curcio, Saddek Mohand-Saïd, José-Alain Sahel, Isabelle Audo, Mustapha Benchaboune, Angela J. Cree, Christina A. Rennie, Srinivas V. Goverdhan, Michelle Grunin, Shira Hagbi-Levi, Peter Campochiaro, Nicholas Katsanis, Frank G. Holz, Frédéric Blond, Hélène Blanché, Jean-François Deleuze, Robert P. Igo, Barbara Truitt, Neal S. Peachey, Stacy M. Meuer, Chelsea E. Myers, Emily L. Moore, Ronald Klein, Michael A. Hauser, Eric A. Postel, Monique D. Courtenay, Stephen G. Schwartz, Jaclyn L. Kovach, William K. Scott, Gerald Liew, Ava G. Tan, Bamini Gopinath, John C. Merriam, R. Theodore Smith, Jane C. Khan, Humma Shahid, Anthony T. Moore, J. Allie McGrath, Reneé Laux, Milam A. Brantley, Anita Agarwal, Lebriz Ersoy, Albert Caramoy, Thomas Langmann, Nicole T. M. Saksens, Eiko K. de Jong, Carel B. Hoyng, Melinda S. Cain, Andrea J. Richardson, Tammy M. Martin, John Blangero, Daniel E. Weeks, Bal Dhillon, Cornelia M. van Duijn, Kimberly F. Doheny, Jane Romm, Caroline C. W. Klaver, Caroline Hayward, Michael B. Gorin, Michael L. Klein, Paul N. Baird, Anneke I. den Hollander, Sascha Fauser, John R. W. Yates, Rando Allikmets, Jie Jin Wang, Debra A. Schaumberg, Barbara E. K. Klein, Stephanie A. Hagstrom, Itay Chowers, Andrew J. Lotery, Thierry Léveillard, Kang Zhang, Murray H. Brilliant, Alex W. Hewitt, Anand Swaroop, Emily Y. Chew, Margaret A. Pericak-Vance, Margaret DeAngelis, Dwight Stambolian, Jonathan L. Haines, Sudha K. Iyengar, Bernhard H. F. Weber, Gonçalo R. Abecasis, Iris M. Heid, Iris M. Heid, Bernhard H. F. Weber

**Affiliations:** 1 Institute of Human Genetics, University of Regensburg, Franz-Josef-Strauss-Allee 11, 93053 Regensburg, Germany; 2 Department of Genetic Epidemiology, University of Regensburg, Franz-Josef-Strauss-Allee 11, 93053 Regensburg, Germany; 3 Institute of Medical Microbiology and Hygiene, University of Regensburg, Franz-Josef-Strauss-Allee 11, Regensburg, 93053 Germany

**Keywords:** Age-related macular degeneration, AMD, Genetic risk scores, GRS, Genetic association studies, Complex traits, Shared genetics

## Abstract

**Background:**

Age-related macular degeneration (AMD) is a common condition of vision loss with disease development strongly influenced by environmental and genetic factors. Recently, 34 loci were associated with AMD at genome-wide significance. So far, little is known about a genetic overlap between AMD and other complex diseases or disease-relevant traits.

**Methods:**

For each of 60 complex diseases/traits with publicly available genome-wide significant association data, the lead genetic variant per independent locus was extracted and a genetic score was calculated for each disease/trait as the weighted sum of risk alleles. The association with AMD was estimated based on 16,144 AMD cases and 17,832 controls using logistic regression.

**Results:**

Of the respective disease/trait variance, the 60 genetic scores explained on average 4.8% (0.27–20.69%) and 16 of them were found to be significantly associated with AMD (Q-values < 0.01, *p* values from < 1.0 × 10^–16^ to 1.9 × 10^–3^). Notably, an increased risk for AMD was associated with reduced risk for cardiovascular diseases, increased risk for autoimmune diseases, higher HDL and lower LDL levels in serum, lower bone-mineral density as well as an increased risk for skin cancer. By restricting the analysis to 1824 variants initially used to compute the 60 genetic scores, we identified 28 novel AMD risk variants (Q-values < 0.01, *p* values from 1.1 × 10^–7^ to 3.0 × 10^–4^), known to be involved in cardiovascular disorders, lipid metabolism, autoimmune diseases, anthropomorphic traits, ocular disorders, and neurological diseases. The latter variants represent 20 novel AMD-associated, pleiotropic loci. Genes in the novel loci reinforce previous findings strongly implicating the complement system in AMD pathogenesis.

**Conclusions:**

We demonstrate a substantial overlap of the genetics of several complex diseases/traits with AMD and provide statistically significant evidence for an additional 20 loci associated with AMD. This highlights the possibility that so far unrelated pathologies may have disease pathways in common.

**Electronic supplementary material:**

The online version of this article (doi:10.1186/s13073-017-0418-0) contains supplementary material, which is available to authorized users.

## Background

Age-related macular degeneration (AMD) is the most common cause of vision loss in senior citizens [1–3]. One of the first signs of AMD is the appearance of yellowish drusen between the retinal pigment epithelium (RPE) and Bruch’s membrane. Drusen comprise extracellular deposits of proteins and lipids and predispose individuals to develop the degenerative late-stage form of the disease [4]. Late-stage AMD manifests as geographic atrophy (GA) or neovascular (NV) AMD although both late-stage forms can be present in the same or in different eyes of a single individual (mixed GA + NV). GA affects up to 50% of people with late-stage AMD and is defined as a discrete region of RPE atrophy. NV AMD describes the abnormal growth of leaky blood vessels from the choroid or from within the retina resulting in detachment of the RPE, strong immune cell activation, photoreceptor cell death, and eventually widespread RPE damage. Although vision loss is more rapid in NV AMD, visual acuity can be preserved by anti-angiogenic treatment [5, 6].

Over the past decade, genome-wide association studies (GWAS) have identified a number of single nucleotide variants (SNVs) as well as copy number variations in complement and complement-related genes that are involved in AMD risk [7–12]. Recently, the International AMD Genomics Consortium (IAMDGC) [13] identified 52 independent genetic variants at 34 loci across the genome to be associated with late-stage AMD, explaining up to 50% of the heritability of this disorder.

The genetic risk of an individual to develop a disease can be expressed as a genetic score. One concept to calculate such a score is to count the number of risk-increasing alleles. To account for differences between the effect sizes of genetic variants (i.e. the relative influence of each variant on disease risk), the score can be calculated as the weighted sum of risk increasing alleles using the relative effect size of a variant as weight [14]. Such scores effectively summarize the genetic contribution to diseases or traits and allow evaluation of the genetic risk of other diseases and traits and its correlation with AMD risk.

To evaluate shared genetics between AMD and other complex diseases or disease-relevant traits, we computed genetic scores from published genome-wide significant lead variants for 60 diseases and traits [15–81] and examined their association with AMD using data from a large AMD case-control study including more than 33,000 participants.

## Methods

### Description of dataset

In total, we included data from 16,144 people with late-stage AMD (NV, GA, or both, GA/NV AMD) and 17,832 control individuals without AMD, all unrelated and of European ancestry [13]. Inclusion and exclusion criteria as well as detailed information on ophthalmological grading, quality control of genetic data as well as imputation are given in detail elsewhere [13]. The dataset contained 14,352 men and 19,624 women. Twenty-six studies with different study designs contributed to this dataset, including six population-based studies (2166 cases, 4246 controls).

### Diseases and traits under evaluation

We searched PubMed (www.pubmed.gov) for GWAS of human diseases and traits which included primarily individuals of European descent and publication dates prior to April 2016 (Additional file 1: Figure S1). In addition, we queried GWAS Central (www.gwascentral.org/browser) using the same criteria. GWAS were excluded when no genome-wide significant variants (*p* < 5.00 × 10^–8^ or log10 p < –7.3010) were reported or when relevant data such as effect sizes, effect alleles, or *p* values of association were missing. We also excluded GWAS dealing with diseases and traits mainly attributable to childhood or pregnancy and behavioral/lifestyle traits. In total, we selected 60 human diseases or traits that were eligible for further analysis.

### Calculation of genetic (risk) scores

For each of the 60 diseases/traits, we extracted independent genetic variants associated with each of the diseases/traits from the relevant publication, provided their individual association reached genome-wide significance (p < 5.00 × 10^–8^ or log10 p < –7.3010) (Additional file 1: Figure S1 and Additional file 2: Table S1). SNVs were included with an imputation quality > 0.3, but only those for which we could determine the risk increasing (effect) allele and the associated effect size. Structural variants (e.g. deletions or duplications) were included if they could be imputed reliably (imputation quality > 0.3) into the IAMDGC dataset. Otherwise, if available, proxy variants with R^2^ > 0.95 with sufficient imputation quality were chosen. In this analysis, a locus region was defined by a genome-wide significant variant and variants within ± 1 Mbp. For each locus, only the lead variant (i.e. the variant with the smallest *p* value for association) was included to represent the relevant disease-/trait-associated haplotype. In a few cases with multiple independent variants reported within a locus, we included all of these. We excluded diseases/traits with less than three genome-wide significant variants published. To account for differences in effect sizes, we extracted the relevant measure of the effect for each of the identified variant (log odds ratio [LOR] for binary outcome, log hazard ratio [HR] for survival, or slope for continuous outcome) from published sources for the respective lead variant (Additional file 2: Table S1).

For each disease/trait, the genetic score was calculated as reported in [14] with slight modifications. Briefly, the number of risk increasing alleles, each weighted (multiplied) by the respective effect size (LOR, log HR, or linear regression slope), were counted and the total divided by the average weight. Thus, an individual with a genetic score that is one unit larger than the genetic score of another individual has one additional risk-increasing allele with average effect size. An individual with a genetic score of 50 would have 50 “average” risk-increasing alleles. In addition to the 60 genetic scores for the selected diseases/traits, we computed the genetic score for AMD based on 52 identified independent AMD variants [13].

The diseases/traits included in the study and the respective publications used to extract the variants and effect sizes are listed in Additional file 2: Table S1. The variants of the traits that were included in the analysis and further information on these variants are listed in Additional file 3: Table S2.

### Correlation between genetic scores and variance explained

We computed the correlation coefficient between selected genetic scores in R [82]. The results were plotted with the function heatmap2 from the gplots package [83] using a diverging color palette implemented in the package RColorBrewer [84]. In addition, we estimated the disease/trait variance explained by each variant used to calculate the individual genetic score. In case the disease/trait is dichotomous (e.g. coronary artery disease or psoriasis [PSO]), we extracted the relevant ORs and allele frequencies and calculated the variance explained using a liability threshold model [85]. In case a disease/trait is continuous (e.g. body mass index, height), we calculated the variance explained directly from the respective linear slopes and standard errors.

### Association of the genetic scores with AMD

To understand the role of the genetic background of each of the 60 diseases/traits for AMD, we conducted association analyses for each of the 60 genetic scores with AMD using logistic regression. Each model was adjusted for DNA source (whole genome amplification: yes/no) and principal components to control for potential subpopulations. Additionally, adjustments were done for age and gender [13].

To account for the multiple association testing of the 60 genetic scores, which were correlated due to shared variants or loci, we controlled the false discovery rate (FDR) to be smaller than 1% [86].

The strength of association of genetic scores and variants are reported as the log odds ratio (LOR). The OR depicts the AMD risk increase per unit increase in the genetic score that is the AMD risk increase per one additional risk allele with average effect size.

Additional association testing was performed by subgroups, separately for participants at higher age versus lower age (cutoff: age 75 years), for men and women, and by disease subtype (restricting the cases to GA or to NV/mixed GA + NV using the same controls). For the ocular specific diseases/traits, we additionally restricted the analysis to individuals from population-based studies to avoid possible confounding effects.

### Identification of novel AMD-associated variants and loci

Given the substantial overlap of genetic disease/trait scores with AMD, we reasoned that shared pathways exist and that there might be even more AMD variants among those associated with other diseases/traits. Therefore, we computed the association with AMD for each variant used to compute any of the 60 genetic scores applying logistic regression adjusted for age, gender, DNA source, and the first two principle components. We controlled the FDR to be smaller than 1%. Variants located in one of the 34 known AMD-associated loci [13] were considered to be known variants. To substantiate the independence of the selected variants, we additionally conducted the analyses adjusting for all of the 52 independent AMD-associated variants.

### Pathway enrichment analysis based on novel and known AMD risk variants

To derive information on potential genes influenced by the observed association signal, we extracted all genes of a region around the respective variant. Here, we used the same locus definition as previously reported [13] (most distant variant with R^2^ > 0.5 in a region ± 100 Kbp). In addition, we added the genes from 34 known AMD loci [13]. The resulting gene list was subjected to pathway enrichment analysis using INRICH [87]. We queried significantly enriched KEGG, GO, and Reactome pathways and required at least four genes of the gene list to be present in the respective pathway. The FDR was controlled at 1%.

### Annotation of novel associated and linked variants

We extracted the position of novel associated variants as well as their correlated variants (R^2^ > 0.5) and used the Variant Effect Predictor on www.ensembl.org to find variants in the coding region of a gene [88].

## Results

### Selection of variants and computation of genetic scores

We extracted 1876 independent genome-wide significant variants for 60 diseases/traits from previously published sources (Additional file 1: Figure S1 and Additional file 2: Table S1). For each disease/trait, we computed a weighted genetic score in our 16,144 cases with late-stage AMD (NV AMD, GA, or both NV/GA AMD) and 17,832 controls without signs of early-stage or late-stage AMD. Additionally, we computed a genetic score for AMD based on the 52 identified AMD variants [13].

### Pairwise correlation of genetic scores

**Fig. 1:**
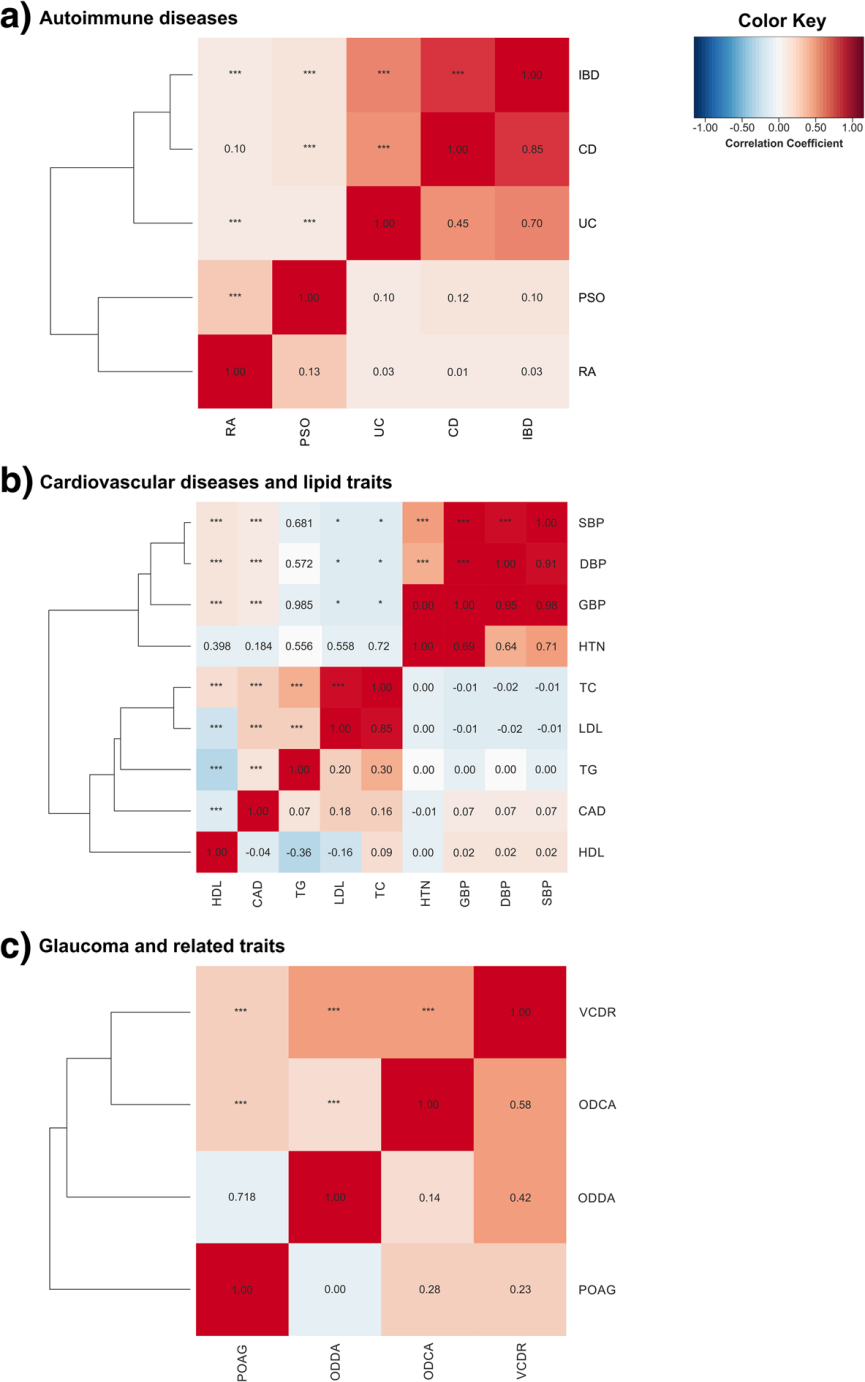
Pairwise correlation between selected genetic scores. The *color key* represents the strength of the correlation between pairs of genetic scores, estimated as the correlation coefficient. The numbers on the bottom half of the graph indicate the correlation coefficient. The numbers in the top half indicate the statistical significance of the observed correlation coefficient (*Q-value < 0.01, **Q-value < 0.001, ***Q-value < 0.0001). The abbreviations used for each trait are listed in Additional file 2: Table S1

To understand the dependencies between the genetic scores, pair-wise correlation coefficients of the scores were investigated (Fig. 1). We observed plausible correlations between genetic scores particularly of traits related to autoimmunity (inflammatory bowel disease, Crohn’s disease and ulcerative colitis, rheumatoid arthritis (RA), PSO; Spearman correlation coefficient r in the range of 0.01–0.85), cardiovascular disease risk, and lipid levels in blood (blood pressure, coronary artery disease, hypertension, high density lipoprotein (HDL), low density lipoprotein (LDL), total cholesterol and total glycerol; r in the range of -0.36–0.98). Interestingly, the score for the glaucoma-related trait “optic disc/disc area” (ODDA) was not correlated to the genetic score of primary open angle glaucoma (POAG, r = 0.00), indicating that both phenotypes share no known genetic overlap. In summary, these correlations are in line with known relationships of the respective diseases and traits [24, 40] and with previously published correlations between genetic loci of diseases/traits [89]. On average, the 60 genetic scores explain 4.81% of the variance of each disease/trait.

### Association of genetic scores with AMD

**Fig. 2:**
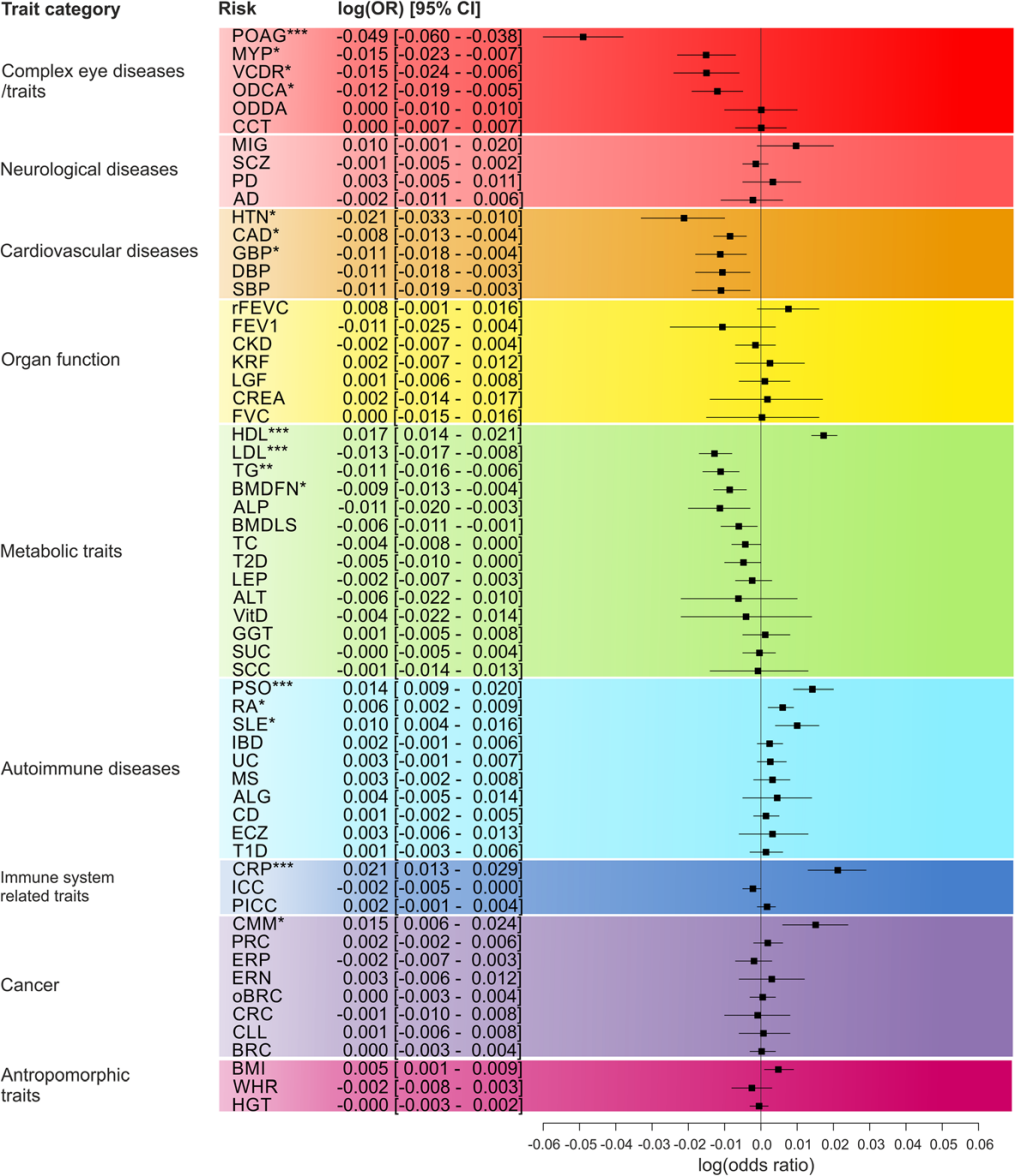
Association of 60 genetic scores with AMD. Logistic regression models, adjusted for age, gender, the first two principle components computed from the genotypes as well as DNA source were fitted for 60 genetic scores of selected complex diseases and traits. LOR (*squares*) and 95% confidence intervals (*horizontal lines*) obtained for each genetic score are plotted. *Q-value < 0.01, **Q-value < 0.001, ***Q-value < 0.0001

**Fig. 3:**
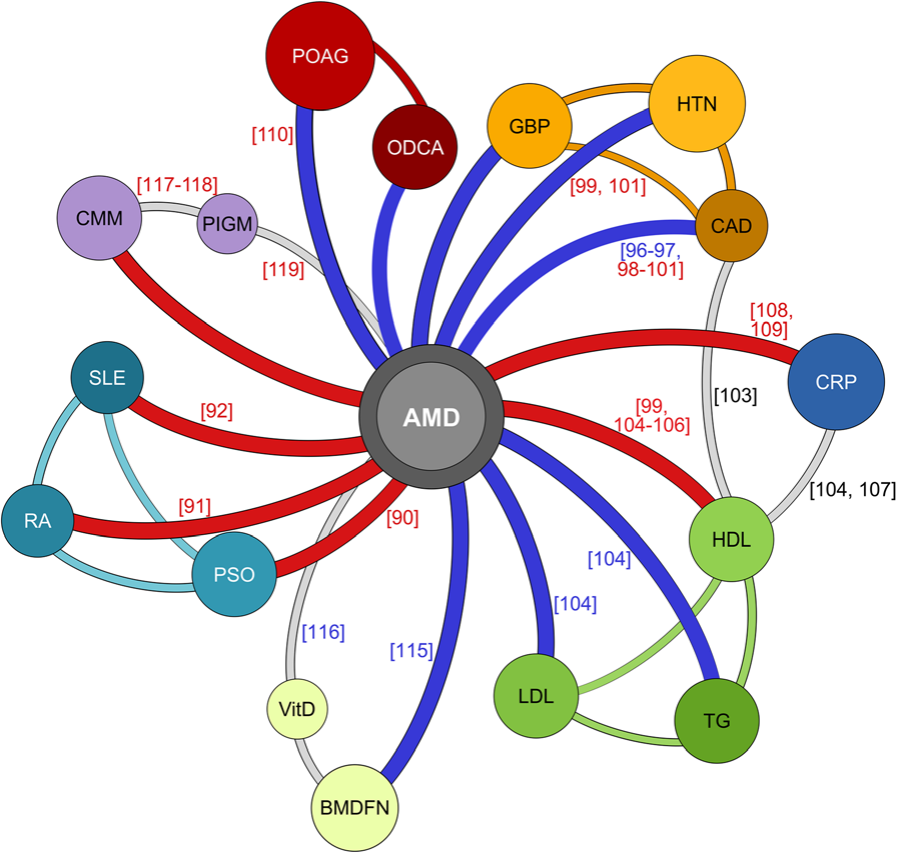
Relationship between complex diseases/traits and AMD based on significant genetic score associations. *Nodes* represent diseases or traits and are colored according to uniform color scheme (see also Fig. 2 and Additional file 2: Table S1). The size of each node represents the effect size of the association with AMD. Diseases and traits within distinct disease categories (see also Fig. 2) are connected with *lines* colored according to the respective disease category. *Lines* connecting AMD and diseases/traits indicate the direction of the association with *red lines* indicating an adverse association and *blue lines* representing protective associations. *Gray lines* depict interactions according to literature which could not be confirmed by genetic scores or were not investigated within this study. The numbers in *brackets* indicate the references which either support or dispute the respective interaction. The colors of the numbers indicate whether the cited literature reported an adverse (*red*) or a protective (*blue*) interaction. In case a finding is novel, no literature reference is presented on a connection between nodes

Next, we investigated the association of the 60 calculated genetic scores with AMD using logistic regression models, adjusted for age, gender, DNA source, and the first two principle components. We found a statistically significant association for the genetic scores for 16 diseases/traits with AMD when controlling the FDR to be at 1% (Figs. 2 and 3). Three genetic scores related to autoimmunity (PSO, RA, and systemic lupus erythematosus [SLE]) were associated with increased risk for AMD, suggesting that participants at increased risk for autoimmune-related diseases are at higher risk for AMD. The remaining seven autoimmune-related genetic scores were consistent in trend (i.e. higher genetic scores are associated with higher AMD risk), although they failed to reach statistical significance. Similarly, we found increased AMD risk for higher genetic scores for elevated C-reactive protein (CRP).

Interestingly, our findings revealed that participants with higher scores for cardiovascular diseases such as hypertension (HTN) or coronary artery disease (CAD) are at decreased risk for AMD. In line with this, participants with more alleles that increase blood pressure have a reduced risk of developing AMD. Furthermore, several scores related to adverse lipid levels in blood are associated with decreased AMD risk: participants with lower HDL genetic scores and higher LDL and total glycerol genetic scores were found to have decreased risk of AMD (Fig. 2). Participants with more alleles for higher bone-mineral density levels in the femoral neck (BMDFN) have decreased risk for AMD. Although we did not find a consistent trend for the association of genetic scores of various types of cancer and AMD, we found that participants at higher genetic risk for cutaneous malignant melanoma (CMM) have an increased risk for AMD. This association can, however, not be attributed to a single variant in the CMM score, since none of the 20 variants used to calculate the score was individually found to be significantly associated with AMD.

Next, we investigated the association of genetic scores of eye-related diseases/traits with AMD risk (Fig. 2, Additional file 1: Figure S2). We found a highly significant association of the myopia genetic score with AMD revealing a strong protective effect. Similarly, AMD patients have fewer risk alleles generally implicated in POAG and related traits of the optic disc area (optic disc cup area [ODCA] and vertical cup to disc ratio [VCDR]). Since the controls of our study were largely recruited in ophthalmologic clinics, it is possible that the association of glaucoma genetic scores can be explained by an enrichment of individuals with glaucoma in our controls. We therefore investigated the association of the glaucoma and related genetic scores restricted to study participants from population-based (cross-sectional) studies. This analysis included 6412 individuals and revealed a consistent protective association of POAG and ODCA with AMD (Additional file 1: Figure S2). Of note, the association of VCDR and myopia (MYP) was markedly weaker in individuals recruited in population-based studies (Additional file 1: Figure S2).

### Association of candidate variants with AMD

Given the association of genetic scores for several diseases with AMD risk indicating an overlap of various disease etiologies with AMD, we were interested to search for novel AMD-associated variants among those identified. Therefore, we analyzed the 1824 variants used for the calculation of the 60 scores for potential association with AMD risk. To account for multiple testing, we again controlled the FDR at 1%. Consequently, 31 novel variants were found to be associated with AMD risk (Q-value < 0.01, p values 1.07 × 10^–7^ to 3.0 × 10^–4^; Additional file 3: Table S2). Moreover, this association was conditioned for 52 AMD-associated risk variants to exclude the significant association signals which may be due to linkage to any of the 52 known AMD associated variants. Following this adjustment, 28 variants remained significantly associated with AMD risk (Q-value < 0.01, Table 1).

**Table 1:** Single variant analysis of variants significantly associated with AMD risk

						Original study		Association with AMD						Frequency in		
Variant	Chromo some	Position [hg19]	Phenotype	Locus name	Locus boundary in 1 M bp [hg19]	Effect allele^a^	Effect size^a^	Odds ratio	95% CI^b^	*p* value raw	*p* value adj^c^	Q-value raw	Q-value adj^c^	Controls	Cases	Imputation quality
rs7523273	1	207977083	SCZ	*CD46/CR1L*	207.8–208.1	A	1.063 (OR)	1.078	1.042-1.116	1.37E-05	2.51E-05	0.0009	5.91E-05	0.667	0.681	0.972
rs1550094	2	233385396	MYP	*PRSS56*	233.3–233.5	G	1.087 (HR)	1.067	1.030-1.106	0.0003	0.0019	0.0093	0.0021	0.290	0.304	0.867
rs9844666	3	135974216	HGT	*STAG1*	135.5–136.9	G	1.024 (SL)	1.079	1.039-1.120	6.70E-05	0.0002	0.0029	0.0003	0.753	0.768	0.999
rs7432375	3	136288405	SCZ	*STAG1*	135.5–136.9	G	1.073 (OR)	0.942	0.911-0.973	0.0003	0.0004	0.0095	0.0005	0.605	0.593	0.999
rs13091182	3	141133960	MYP	*ZBTB38*	141–141.3	G	1.064 (OR)	0.929	0.898-0.960	1.65E-05	5.65E-06	0.0010	2.26E-05	0.668	0.651	0.996
rs3774959	4	103511114	UC	*NFKB1*	103.3–103.6	A	1.119 (OR)	0.928	0.898-0.959	1.11E-05	4.44E-07	0.0007	7.71E-06	0.357	0.341	0.964
rs2277027	5	156932376	LGF	*ADAM19*	156.8–157.1	A	1.462 (SL)	1.096	1.059-1.133	1.31E-07	2.75E-06	1.50E-05	1.28E-05	0.651	0.667	1.000
rs2814982	6	34546560	TC	*C6ORF106*	34.4–34.9	C	1.045 (SL)	1.118	1.061-1.178	2.67E-05	4.82E-05	0.0014	0.0001	0.889	0.898	0.980
rs2207139	6	50845490	BMI	*TFAP2B*	50.7–51	G	1.046 (SL)	0.921	0.883-0.961	0.0001	0.0004	0.0058	0.0006	0.179	0.165	0.997
rs11776767	8	10683929	TG	*PINX1*	10.5–10.8	C	1.022 (OR)	0.936	0.905-0.967	8.83E-05	0.0089	0.0037	0.0089	0.380	0.370	0.999
rs3217992	9	22003223	CAD	*CDKN2B*	21.9–22.2	T	1.160 (OR)	0.914	0.884-0.945	1.07E-07	1.18E-05	1.35E-05	3.67E-05	0.394	0.375	1.000
rs7865618	9	22031005	ODCA; VCDR	*CDKN2B*	21.9–22.2	A	1.023 (SL)	0.937	0.907-0.968	9.23E-05	6.12E-05	0.0037	0.0001	0.593	0.579	1.000
rs7866783	9	22056359	POAG	*CDKN2B*	21.9–22.2	G	1.451 (OR)	0.936	0.906-0.967	6.67E-05	2.53E-05	0.0029	5.91E-05	0.594	0.579	0.995
rs11191548	10	104846178	BP	*NT5C2*	104.4–105.2	T	2.989 (SL)	0.902	0.853-0.954	0.0003	0.0012	0.0092	0.0014	0.914	0.906	1.000
rs11191560	10	104869038	BMI	*NT5C2*	104.4–105.2	C	1.031 (SL)	1.110	1.049-1.173	0.0003	0.0012	0.0087	0.0014	0.086	0.094	0.996
rs634552	11	75282052	HGT	*SERPINH1*	75.2–75.4	T	1.040 (SL)	1.120	1.069-1.173	2.08E-06	1.47E-05	0.0002	4.11E-05	0.130	0.144	0.999
rs11830103	12	123823546	HGT	*SBNO1*	123.3–124	G	1.036 (SL)	1.082	1.040-1.125	9.13E-05	0.0028	0.0037	0.0029	0.205	0.216	1.000
rs4901977	14	60789176	VCDR	*SIX6*	60.7–61.3	T	1.011 (SL)	0.927	0.895-0.959	1.56E-05	8.40E-06	0.0010	2.94E-05	0.319	0.306	0.996
rs2093210	14	60957279	HGT	*SIX6*	60.7–61.3	C	1.033 (SL)	0.920	0.890-0.950	5.00E-07	1.37E-06	4.34E-05	7.71E-06	0.417	0.401	0.997
rs33912345	14	60976537	POAG	*SIX6*	60.7–61.3	C	1.320 (OR)	0.919	0.889-0.949	3.51E-07	1.19E-06	3.21E-05	7.71E-06	0.413	0.397	1.000
rs10483727	14	61072875	ODCA	*SIX6*	60.7–61.3	T	1.026 (SL)	0.915	0.886-0.946	1.11E-07	1.06E-06	1.35E-05	7.71E-06	0.414	0.396	0.999
rs1555399	14	67984370	PD	*PLEKHH1*	67.9–68.1	T	1.115 (OR)	0.929	0.899-0.959	6.27E-06	0.0002	0.0004	0.0003	0.517	0.502	0.997
rs11627032	14	93104072	BRC	*RIN3*	93–93.2	T	1.060 (OR)	1.070	1.032-1.109	0.0002	0.0014	0.0086	0.0015	0.728	0.739	0.931
rs1378942	15	75077367	BP	*CSK*	74.9–75.3	C	1.846 (SL)	0.918	0.887-0.950	8.52E-07	1.38E-06	7.06E-05	7.71E-06	0.345	0.328	1.000
rs2290400	17	38066240	T1D	*GSDMB*	37.8–38.2	C	1.080 (OR)	1.068	1.034-1.103	5.47E-05	7.29E-05	0.0026	0.0001	0.494	0.508	1.000
rs9303280	17	38074031	ALG	*GSDMB*	37.8–38.2	C	1.070 (SL)	0.941	0.911-0.971	0.0002	0.0001	0.0068	0.0002	0.510	0.497	0.984
rs386000	19	54792761	HDL	*LILRA3*	54.7–54.9	C	1.049 (SL)	1.096	1.053-1.141	6.24E-06	0.0006	0.0004	0.0008	0.199	0.211	0.782
rs1547014	22	29100711	VCDR	*CHEK*	28.5–29.2	C	1.013 (SL)	0.936	0.904-0.969	0.0002	6.78E-05	0.0068	0.0001	0.708	0.695	0.999

^a^Risk-/trait-increasing allele and effect size in the original study (*OR* odds ratio, *SL* slope, *HR* hazard ratio) (see Additional file 2: Table S1 for further details)

^b^95% CI = 95% confidence interval of AMD OR estimate

^c^Association of variant adjusted for 52 AMD-associated risk variants

Next, we extracted the variants correlated to the respective top variant at each locus (R^2^ > 0.5) and annotated these using the Variant Effect Predictor [88]. In total, we identified 12 non-synonymous and 13 synonymous variants (Table 2).

**Table 2:** Coding variants in novel AMD-associated loci

Variant	Top variant	Chromosome	Position [hg19]	r^2^ to top variant	Frequency of variant	Affected gene	Consequence	Phenotype	Locus name	Locus boundary in 1 M bp [hg19]
rs6683902	rs7523273	1	207881557	0.545	0.563	CR1L	p.I455V	SCZ	*CD46/CR1L*	207.8–208.1
rs2796257	rs7523273	1	207890866	0.549	0.434	CR1L	p.L491P	SCZ	*CD46/CR1L*	207.8–208.1
rs1550094	rs1550094	2	233385396	1.000	0.310	PRSS56	p.A30T	MYP	*PRSS56*	233.3–233.5
rs9860801	rs9844666	3	136088038	0.547	0.302	STAG1	p.F403F	HGT	*STAG1*	135.5–136.9
rs1052620	rs9844666	3	136574521	0.956	0.189	SLC35G2	p.L407L	HGT	*STAG1*	135.5–136.9
rs1422795	rs2277027	5	156936364	0.996	0.357	ADAM19	p.S17G	LGF	*ADAM19*	156.8–157.1
rs943037	rs11191548	10	104835919	0.988	0.088	CNNM2	p.A770A	BP	*NT5C2*	104.4–105.2
rs584961	rs634552	11	75277628	0.625	0.888	SERPINH1	p.L78L	HGT	*SERPINH1*	75.2–75.4
rs12811109	rs11830103	12	123471094	0.838	0.216	PITPNM2	p.H1205H	HGT	*SBNO1*	123.3–124
rs1051431	rs11830103	12	123645803	0.878	0.768	MPHOSPH9	p.Y935H	HGT	*SBNO1*	123.3–124
rs6488868	rs11830103	12	123799974	0.756	0.722	SBNO1	p.G1022G	HGT	*SBNO1*	123.3–124
rs1060105	rs11830103	12	123806219	0.988	0.223	SBNO1	p.S729N	HGT	*SBNO1*	123.3–124
rs61388686	rs11830103	12	123810873	0.591	0.664	SBNO1	p.I567I	HGT	*SBNO1*	123.3–124
rs12322888	rs11830103	12	123825559	1.000	0.225	SBNO1	p.K209K	HGT	*SBNO1*	123.3–124
rs1254319	rs10483727	14	60903757	0.613	0.301	C14orf39	p.L524F	ODCA	*SIX6*	60.7–61.3
rs33912345	rs10483727	14	60976537	0.978	0.597	SIX6	p.H141N	ODCA	*SIX6*	60.7–61.3
rs117068593	rs11627032	14	93118229	0.565	0.187	RIN3	p.R279C	BRC	*RIN3*	60.7–61.3
rs2470890	rs1378942	15	75047426	0.843	0.596	CYP1A2	p.N516N	BP	*CSK*	74.9–75.3
rs4886615	rs1378942	15	75131661	0.553	0.691	ULK3	p.A302A	BP	*CSK*	74.9–75.3
rs907092	rs2290400	17	37922259	0.761	0.473	IKZF3	p.S399S	T1D	*GSDMB*	37.8–38.2
rs11557466	rs2290400	17	38024626	0.792	0.472	ZPBP2	p.L7L	T1D	*GSDMB*	37.8–38.2
rs11557467	rs2290400	17	38028634	0.924	0.508	ZPBP2	p.S55I	T1D	*GSDMB*	37.8–38.2
rs10852935	rs2290400	17	38031674	0.792	0.472	ZPBP2	p.C174C	T1D	*GSDMB*	37.8–38.2
rs2305480	rs2290400	17	38062196	0.815	0.466	GSDMB	p.P302S	T1D	*GSDMB*	37.8–38.2
rs2305479	rs2290400	17	38062217	0.948	0.502	GSDMB	p.G295R	T1D	*GSDMB*	37.8–38.2

Finally, we defined AMD associated loci around the top variants with the boundaries comprising the most distant variant with R^2^ > 0.5 and added a margin of 100 Kbp to both boundaries. In total, the 28 novel variants defined 20 loci associated with late-stage AMD (Table 1) and thus implicated potential novel genes involved in disease risk. We extracted the genes located in the 20 novel and 34 known loci [7] and used INRICH to perform pathway enrichment analysis. This approach strengthens the notion that complement activation is the main pathway involved in AMD risk (Reactome NCBI *Regulation of complement cascade*: Q-value = 0.0006, GO *Regulation of complement activation*: Q-value = 0.002). No other pathway reached the significance threshold (Q-value < 0.01, Additional file 4: Table S3).

## Discussion

Here, we show an association of genetic scores of 16 different diseases/traits with late-stage AMD. Most notably, we found genetic scores of autoimmune diseases (PSO, RA, and SLE), cardiovascular health (CAD, general blood pressure [GBP], HTN) and lipid levels (HDL, LDL, and triglyceride [TG]) to be associated with AMD. Remarkably, the genetic score for BMDFN as well as the genetic score for CMM were also associated with AMD. We also found that several genetic scores related to other ocular diseases (POAG, VCDR, MYP, and ODCA) are associated with AMD risk. Under the assumption that a genetic score summarizes the known genetic factors for a disease/trait, we conclude that these 16 diseases/traits share etiological properties with AMD.

Our findings point to two major areas of interest. First, we demonstrate that genetic scores related to autoimmunity are associated with AMD with adverse effects, in agreement with the observation that the presence of either PSO, RA, or SLE resulted in a higher risk for AMD [90–92]. Overall, all of the autoimmunity-related scores were higher in AMD patients than in controls. This strengthens the notion that AMD greatly overlaps with or possibly is an autoimmune-related disease. It remains to be seen whether these patients at risk for AMD might profit from anti-inflammatory or immuno-suppressive medication [93–95].

Second, individuals with increased genetic risk for cardiovascular disease and related traits have a lower risk for AMD, which could potentially be explained by a survival bias since our cases are on average two years older than our controls. Nevertheless, by adjusting for gender and age, we should be able to account for the findings. Still, we find a strong association of cardiovascular disease traits with AMD. Such a protective effect of cardiovascular-related genetic scores for AMD is in line with previous results [96, 97], despite disparate reports by other groups [98–101]. The latter discordance may be explained by factors other than genetic influences on cardiovascular disease risk and such factors may be elevated in late-stage AMD patients. For example, it has long been recognized that HDL plays a crucial role in preventing cardiovascular disease and is believed to be neuroprotective while reducing the risk for other neuropathies, e.g. Alzheimer’s disease [102] due to anti-inflammatory and anti-oxidant properties [103]. According to our findings, AMD patients should have higher HDL, lower LDL, and lower total glycerol levels in their serum compared with controls, well in agreement with published data [104–106]. On another note, high levels of HDL and low total glycerol have been correlated with increased complement activation [104, 107]. This could explain the observed association where variants causing high HDL levels are also associated with increased CRP levels in serum [104], additionally increasing complement activation levels [55]. This is in line with the observation that elevated CRP levels are a risk factor for AMD [108, 109]. The risk for AMD may be increased by the same factors due to increased complement activation.

The association of genetic scores of ocular traits with AMD requires a more in-depth consideration. While the genetic scores for central cornea thickness (CCT) and ODDA are not associated with AMD, the remaining genetic scores associated with glaucoma (POAG, ODCA, and VCDR) as well as the genetic score for myopia were protective for AMD. Under the assumption that our controls are enriched for glaucoma and myopia cases, a conceivable expectation for a hospital-based recruitment of controls, the identified association could be explained but would render this finding an artefact. Controls are often enrolled as patients visiting the clinic for reasons other than AMD and may thus be enriched for other prevalent ocular diseases. Furthermore, AMD cases with myopia may be less frequently recruited since grading of AMD can be difficult in the presence of high myopia. Consequently, there may be fewer myopia patients in the patient cohort. On the other hand, population-based studies, i.e. studies randomly recruiting patients and controls in a community setting, should not be compromised by an enrichment of any ocular disease unless they share a similar genetic or non-genetic risk. Testing our dataset for this possibility showed that the association of the genetic scores in the population-based studies remained unchanged when comparing to the entire data including case-control studies with the exception of the VCDR and MYP genetic scores. From this, we conclude that AMD patients indeed have a genetically reduced risk to develop open angle glaucoma, although a smaller study found an adverse relationship between AMD and POAG using summary statistics [110]. Conversely, we found no association of the myopia genetic score with AMD in our dataset in the population-based studies, in line with previous reports [111–114].

A surprising finding was the association of bone-mineral density genetic scores with AMD. Both scores of bone-mineral density (in the femoral neck and in the lumbar spine) are nominally significant, suggesting a protective effect for AMD in individuals having a higher bone-mineral density. Interestingly, vitamin D deficiency was linked to incident AMD in the CARED study [115] and bone-mineral density can be increased with vitamin D supplementation [116]. Of note, neither the genetic score of serum calcium concentration nor vitamin D concentration was found to be significantly associated with AMD. Nevertheless, these findings could point to future studies that explore vitamin D and calcium supplementation to prevent AMD.

The significant association of CMM with AMD is not due to a single variant associated with both diseases, as none of the 20 variants used for the calculation of the genetic score by itself was significantly associated with AMD. The effect seems to result from an accumulation of all 20 variant effect sizes (mean LOR = 0.018). A possible explanation may be that pigmentation plays a role in both diseases. CMM is a cancer type affecting the skin and associations with different characteristics of pigmentation are described [117]. In general, people with lighter skin have a higher melanoma risk [118]. This is in accordance with observations that Caucasians are more likely to be affected by AMD compared with black individuals [119].

Our candidate variant approach was restricted to variants significantly associated with other diseases/traits and revealed 20 novel AMD-associated loci with 28 AMD associated variants. These variants have been implicated in other diseases/traits with genome-wide significance and therefore represent compelling novel findings, although these novel loci do not harbor genes that provide insights into so far unknown AMD-associated pathways. However, the newly identified loci strengthen the notion that AMD disease is extensively related to pathologic complement activation with the discovery that variants in the *CD46/CR1L* locus are significantly associated with AMD. In subsequent studies, in-depth bioinformatics and molecular evaluation of these risk signals need to be performed, particularly in the light of pathways and mechanisms associated with both AMD and the relevant disease/trait.

The results of this study are in accordance with the antagonistic pleiotropy theory of aging [120], which states that many pleiotropic genetic factors are beneficial at younger ages (by either increasing fecundity or survival) while possibly unfavorable later in life by influencing senescence and thus age-related disease processes [121]. We speculate that this could also be true for pleiotropic variants associated with AMD. For instance, increased immune activity could be advantageous at younger ages by reducing the risk for infections, but could ultimately lead to self-tissue damage causing autoimmune disease and late-stage AMD. Similarly, increased HDL and lower LDL levels result in improved cardiovascular health, which is an important factor to survive to old age. However, these same processes can cause late-stage AMD and might persist in populations due to a lack of negative selection in the elderly. As a consequence, our findings challenge the prospects of gene/genome manipulations to target a selected complex disease or trait, since seemingly beneficial genetic manipulations targeting a specific disease might in fact result in a seemingly unrelated disease at old age or accelerate aging in consequence.

## Conclusions

Our findings suggest a substantial overlap of the genetics of autoimmune diseases, cardiovascular traits, lipid metabolism, cancer and metabolic traits as well as other eye-related diseases and traits with AMD. Investigating the association of variants associated with other diseases proves worthwhile to identify novel AMD risk variants and further implicates the complement system as the major pathway involved in AMD pathology.

## Additional files

Additional file 1: Figures. Supplementary Figures S1 and S2 with Figure Legends. (PDF 1935 kb)

Additional file 2: Table S1. Characteristics of diseases/traits included in the study. (XLSX 22 kb)

Additional file 3: Table S2. Detailed list of the genetic variants extracted from all studies. Contains multiple tabs. (XLSX 671 kb)

Additional file 4: Table S3. Pathway enrichment analysis results and parameters. (XLSX 14 kb)
