# Supplementary material for: Genetic pleiotropy between age-related macular degeneration and 16 complex diseases and traits
Source: Genome Med. 2017 Mar 27;9:29. doi: 10.1186/s13073-017-0418-0 (PMC5368911; doi:10.1186/s13073-017-0418-0)

Additional File 1 – Supplementary Figures

Supplementary Figure S1. Workflow to select publicly available GWAS data for association analyses in late stage AMD. Literature search was conducted using various databases with defined criteria for study inclusion. Features of genome-wide significant variants were extracted from the literature (**Additional file 3: Supplementary Table S2**) and the genotypes of the respective variants were retrieved from the IAMDGC dataset. Genotypes passed quality control measures and were arranged according to the risk/trait increasing alleles of the respective disease/trait (**Additional file 3: Supplementary Table S2**). Based on these genotypes, a genetic score was calculated for each disease/trait. The genetic scores were validated by pairwise linear regression between genetic scores in distinct trait clusters (**Figure 1**) and the association of genetic scores with AMD was assessed using multivariate logistic regression (**Figure 2** and **Additional file 2: Supplementary Table S1**). In addition, we investigated the association of 1,824 variants used for genetic score calculation with AMD (**Table 1**). Novel, pleiotropic AMD loci were defined (**Table 1** and **Table 2**) and analyzed using pathway enrichment analysis (**Additional file 4: Supplementary Table S3**).

Supplementary Figure S2. Association analysis of genetic (risk) scores with AMD. Logistic regression models, adjusted for age, gender, the first two principle components computed from the genotypes as well as DNA source were fitted for groups of individuals stratified by age, gender and disease subtype. For disease subtype specific analysis, genetic scores of AMD patients with early AMD, GA, NV or both late stage forms (mixed GA&NV) were compared to the genetic scores computed in all controls. For the gender and age specific analyses, both, cases and controls were

stratified accordingly and analyzed. For the ocular specific diseases/traits, models in individuals recruited in population based studies were fitted to account for confounding effects of the hospital based recruitment strategy. The number of samples included in each analysis is indicated by the size of the rectangles. Log odds ratios and 95% confidence intervals obtained for each genetic score are plotted.

Supplementary Figure S1. Workflow to select publicly available GWAS data for association analyses in late stage AMD.

Search for GWAS data from published sources

Databases (GWAS Catalog, GWAS Central and PubMed keyword searches for „GWAS autoimmune“, „GWAS cancer“, etc.
Criteria included:
- European population/trans-ethnic population including European individuals
- at least 3 genome-wide significant variants per data set ($p < 5x10^{-8}$)
- clearly defined risk increasing alleles and corresponding GWAS results

Extraction of GWAS variants

For each genome-wide significant variant the following parameter was extracted (see also **Additional file 3: Supplementary Table S2**):
- Identifier (rs-ID from dbSNP)
- Genomic location (chromosome and position)
- Effect increasing and reference allele
- Allele frequency in Europeans
- Strand orientation
- Effect size given as OR/HR/beta estimate and 95% confidencence intervals

Variants in the IAMDGC dataset

- Imputed genotype extraction of the respective variants from the IAMDGC dataset (**Additional file 3: Supplementary Table S2**)
- Comparison of allele frequencies and strand orientation
- Exclusion of variants that could not reliably be matched in the IAMDGC dataset (**Additional file 2: Supplementary Table S1**)
- Arranging of variants according to risk/trait increasing alleles of the respective disease/trait

Genetic scores

Calculation of genetic scores per disease/trait in AMD patients (n=16,144) and controls (n=17,832)

Validation

Validation of genetic scores by a pairwise linear regression model between genetic scores in three trait clusters (**Figure 1**)

Association

Association of genetic scores with AMD using multivariate logistic regression models (**Figure 2** and **Additional file 2: Supplementary Table S1**)

GWAS variants

Association testing of 1,824 GWAS variants with AMD risk using logistic regression (**Table 1**).
Criteria for novel AMD variants:
- Exclusion of variants in known AMD loci
- Adjustment for AMD genetic score to account for known variance
- Adjustment for multiple testing

Novel loci

Significant variants defined novel, pleiotropic loci (**Table 1** and **Table 2**):
- Locus definition: $r^2 > 0.5$ to significant variant ± 100kbp
- Merging of overlapping loci

Analysis of novel AMD loci

- Pathway enrichment analysis of new and previously reported AMD risk loci using INRICH (**Additional file 4: Supplementary Table S3**)
- Annotation of significant variants and linked variants ($r^2 > 0.5$) to detect variants in coding exons

Supplementary Figure S2. Association analysis of genetic (risk) scores with AMD in various groups.

CCT Genetic Risk Scores in AMD Patients

Analysis	log(OR) [95% CI]	N
unadjusted	-0.001 [-0.008 - 0.005]	33976
adjusted	0.000 [-0.007 - 0.007]	33976
<75 years	0.002 [-0.008 - 0.012]	16968
>75 years	-0.003 [-0.013 - 0.008]	16650
male only	-0.002 [-0.013 - 0.009]	14352
female only	0.002 [-0.008 - 0.011]	19624
GA	-0.004 [-0.016 - 0.008]	21067
CNV	0.002 [-0.006 - 0.010]	28581
GA&CNV	-0.003 [-0.018 - 0.011]	19992
early AMD	-0.004 [-0.013 - 0.005]	24489

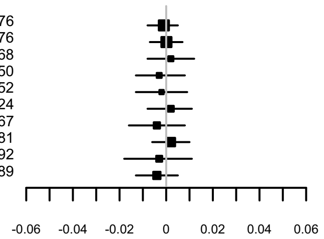

ODDA Genetic Risk Scores in AMD Patients

Analysis	log(OR) [95% CI]	N
unadjusted	-0.001 [-0.010 - 0.008]	33976
adjusted	0.000 [-0.010 - 0.010]	33976
<75 years	-0.001 [-0.015 - 0.014]	16968
>75 years	0.000 [-0.014 - 0.014]	16650
male only	0.011 [-0.004 - 0.026]	14352
female only	-0.008 [-0.021 - 0.005]	19624
GA	0.012 [-0.004 - 0.029]	21067
CNV	-0.002 [-0.013 - 0.009]	28581
GA&CNV	-0.003 [-0.023 - 0.016]	19992
early AMD	0.000 [-0.013 - 0.012]	24489
Glaucoma excl.	0.003 [-0.013 - 0.020]	11316
Pop.-based	-0.004 [-0.027 - 0.020]	6412

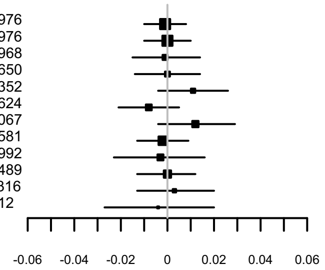

AD Genetic Risk Scores in AMD Patients

Analysis	log(OR) [95% CI]	N
unadjusted	-0.005 [-0.013 - 0.003]	33976
adjusted	-0.002 [-0.011 - 0.006]	33976
<75 years	-0.001 [-0.013 - 0.011]	16968
>75 years	-0.003 [-0.015 - 0.009]	16650
male only	-0.002 [-0.015 - 0.011]	14352
female only	-0.002 [-0.013 - 0.009]	19624
GA	0.000 [-0.014 - 0.014]	21067
CNV	-0.006 [-0.016 - 0.003]	28581
GA&CNV	0.013 [-0.004 - 0.030]	19992
early AMD	0.001 [-0.009 - 0.012]	24489

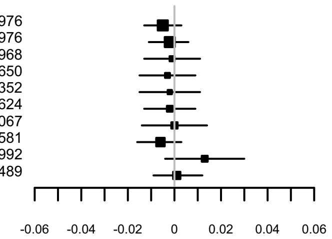

SCZ Genetic Risk Scores in AMD Patients

Analysis	log(OR) [95% CI]	N
unadjusted	-0.002 [-0.005 - 0.002]	33976
adjusted	-0.001 [-0.005 - 0.002]	33976
<75 years	0.000 [-0.005 - 0.004]	16968
>75 years	-0.002 [-0.007 - 0.003]	16650
male only	-0.002 [-0.007 - 0.003]	14352
female only	-0.001 [-0.005 - 0.004]	19624
GA	-0.002 [-0.007 - 0.004]	21067
CNV	-0.001 [-0.004 - 0.003]	28581
GA&CNV	-0.005 [-0.012 - 0.002]	19992
early AMD	-0.002 [-0.006 - 0.003]	24489

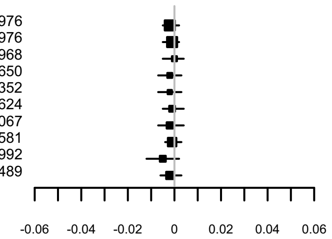

MYP Genetic Risk Scores in AMD Patients

Analysis	log(OR) [95% CI]	N
unadjusted	-0.013 [-0.020 - -0.005]	33976
adjusted	-0.015 [-0.023 - -0.007]	33976
<75 years	-0.015 [-0.027 - -0.003]	16968
>75 years	-0.017 [-0.029 - -0.006]	16650
male only	-0.019 [-0.032 - -0.007]	14352
female only	-0.012 [-0.023 - -0.001]	19624
GA	-0.025 [-0.039 - -0.011]	21067
CNV	-0.012 [-0.021 - -0.003]	28581
GA&CNV	-0.017 [-0.034 - -0.001]	19992
early AMD	-0.004 [-0.014 - 0.007]	24489
Pop.-based	-0.002 [-0.022 - 0.018]	6412

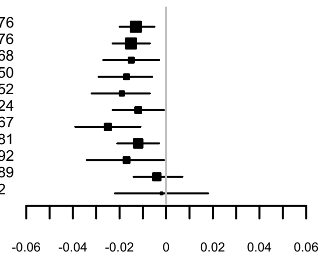

POAG Genetic Risk Scores in AMD Patients

Analysis	log(OR) [95% CI]	N
unadjusted	-0.047 [-0.057 - -0.036]	33976
adjusted	-0.049 [-0.060 - -0.038]	33976
<75 years	-0.048 [-0.065 - -0.032]	16968
>75 years	-0.054 [-0.070 - -0.037]	16650
male only	-0.054 [-0.071 - -0.037]	14352
female only	-0.045 [-0.061 - -0.030]	19624
GA	-0.040 [-0.059 - -0.020]	21067
CNV	-0.050 [-0.062 - -0.038]	28581
GA&CNV	-0.061 [-0.084 - -0.038]	19992
early AMD	-0.051 [-0.066 - -0.037]	24489
Glaucoma excl.	-0.110 [-0.129 - -0.090]	11316
Pop.-based	-0.044 [-0.072 - -0.017]	6412

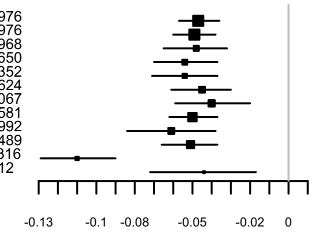

MIG Genetic Risk Scores in AMD Patients

Analysis	log(OR) [95% CI]	N
unadjusted	0.013 [0.003 - 0.023]	33976
adjusted	0.010 [-0.001 - 0.020]	33976
<75 years	0.011 [-0.004 - 0.027]	16968
>75 years	0.006 [-0.009 - 0.021]	16650
male only	0.008 [-0.008 - 0.024]	14352
female only	0.011 [-0.003 - 0.025]	19624
GA	0.009 [-0.009 - 0.027]	21067
CNV	0.011 [0.000 - 0.023]	28581
GA&CNV	0.002 [-0.020 - 0.023]	19992
early AMD	0.001 [-0.012 - 0.014]	24489

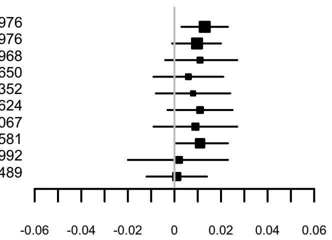

CAD Genetic Risk Scores in AMD Patients

Analysis	log(OR) [95% CI]	N
unadjusted	-0.010 [-0.014 - -0.005]	33976
adjusted	-0.008 [-0.013 - -0.004]	33976
<75 years	-0.011 [-0.018 - -0.004]	16968
>75 years	-0.007 [-0.014 - 0.000]	16650
male only	-0.009 [-0.017 - -0.002]	14352
female only	-0.008 [-0.014 - -0.002]	19624
GA	-0.008 [-0.016 - 0.000]	21067
CNV	-0.009 [-0.014 - -0.003]	28581
GA&CNV	-0.008 [-0.018 - 0.001]	19992
early AMD	-0.006 [-0.012 - 0.000]	24489

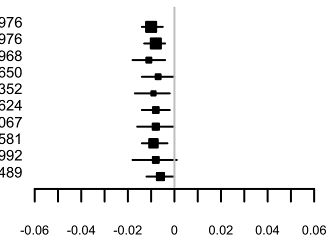

ODCA Genetic Risk Scores in AMD Patients

Analysis	log(OR) [95% CI]	N
unadjusted	-0.011 [-0.018 - -0.004]	33976
adjusted	-0.012 [-0.019 - -0.005]	33976
<75 years	-0.001 [-0.011 - 0.010]	16968
>75 years	-0.022 [-0.032 - -0.011]	16650
male only	-0.014 [-0.025 - -0.003]	14352
female only	-0.011 [-0.020 - -0.001]	19624
GA	-0.005 [-0.018 - 0.007]	21067
CNV	-0.015 [-0.023 - -0.007]	28581
GA&CNV	-0.013 [-0.028 - 0.002]	19992
early AMD	-0.015 [-0.024 - -0.006]	24489
Glaucoma excl.	-0.011 [-0.023 - 0.001]	11316
Pop.-based	-0.015 [-0.032 - 0.002]	6412

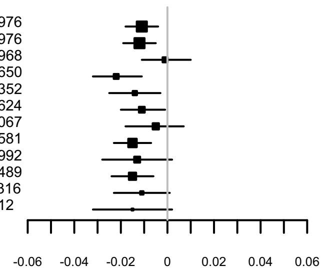

VCDR Genetic Risk Scores in AMD Patients

Analysis	log(OR) [95% CI]	N
unadjusted	-0.015 [-0.024 - -0.007]	33976
adjusted	-0.015 [-0.024 - -0.006]	33976
<75 years	-0.008 [-0.021 - 0.005]	16968
>75 years	-0.022 [-0.035 - -0.009]	16650
male only	-0.015 [-0.028 - -0.002]	14352
female only	-0.016 [-0.027 - -0.004]	19624
GA	-0.004 [-0.019 - 0.011]	21067
CNV	-0.020 [-0.030 - -0.010]	28581
GA&CNV	-0.011 [-0.029 - 0.007]	19992
early AMD	-0.021 [-0.032 - -0.010]	24489
Glaucoma excl.	-0.013 [-0.028 - 0.002]	11316
Pop.-based	-0.002 [-0.023 - 0.020]	6412

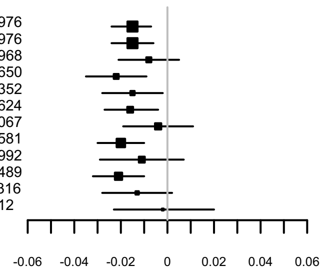

PD Genetic Risk Scores in AMD Patients

Analysis	log(OR) [95% CI]	N
unadjusted	0.001 [-0.006 - 0.008]	33976
adjusted	0.003 [-0.005 - 0.011]	33976
<75 years	0.007 [-0.004 - 0.019]	16968
>75 years	0.001 [-0.010 - 0.012]	16650
male only	0.007 [-0.005 - 0.019]	14352
female only	0.001 [-0.010 - 0.011]	19624
GA	0.007 [-0.007 - 0.020]	21067
CNV	0.000 [-0.008 - 0.009]	28581
GA&CNV	0.013 [-0.003 - 0.029]	19992
early AMD	0.009 [-0.001 - 0.019]	24489

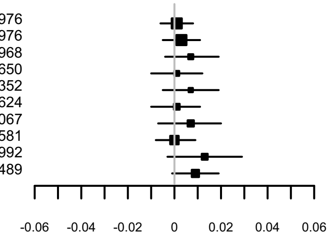

DBP Genetic Risk Scores in AMD Patients

Analysis	log(OR) [95% CI]	N
unadjusted	-0.012 [-0.019 - -0.005]	33976
adjusted	-0.011 [-0.018 - -0.003]	33976
<75 years	-0.013 [-0.023 - -0.002]	16968
>75 years	-0.009 [-0.020 - 0.002]	16650
male only	-0.013 [-0.024 - -0.002]	14352
female only	-0.009 [-0.019 - 0.001]	19624
GA	-0.017 [-0.030 - -0.004]	21067
CNV	-0.010 [-0.018 - -0.001]	28581
GA&CNV	-0.006 [-0.021 - 0.009]	19992
early AMD	0.008 [-0.001 - 0.018]	24489

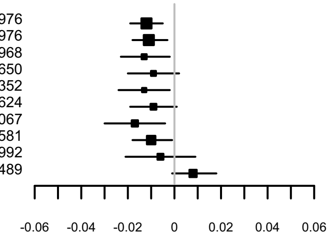

Supplementary Figure S2. continued

GBP Genetic Risk Scores in AMD Patients

Analysis	log(OR) [95% CI]	N
unadjusted	-0.013 [-0.019 - -0.006]	33976
adjusted	-0.011 [-0.018 - -0.004]	33976
<75 years	-0.013 [-0.023 - -0.003]	16968
>75 years	-0.011 [-0.021 - -0.001]	16650
male only	-0.012 [-0.023 - -0.001]	14352
female only	-0.011 [-0.020 - -0.001]	19624
GA	-0.017 [-0.029 - -0.005]	21067
CNV	-0.010 [-0.018 - -0.002]	28581
GA&CNV	-0.009 [-0.024 - 0.005]	19992
early AMD	0.007 [-0.001 - 0.016]	24489

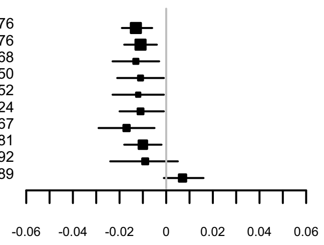

CKD Genetic Risk Scores in AMD Patients

Analysis	log(OR) [95% CI]	N
unadjusted	-0.002 [-0.007 - 0.003]	33976
adjusted	-0.001 [-0.007 - 0.004]	33976
<75 years	0.006 [-0.002 - 0.013]	16968
>75 years	-0.007 [-0.014 - 0.001]	16650
male only	-0.002 [-0.010 - 0.006]	14352
female only	-0.001 [-0.009 - 0.006]	19624
GA	0.002 [-0.007 - 0.011]	21067
CNV	-0.003 [-0.009 - 0.003]	28581
GA&CNV	0.000 [-0.011 - 0.010]	19992
early AMD	-0.001 [-0.007 - 0.006]	24489

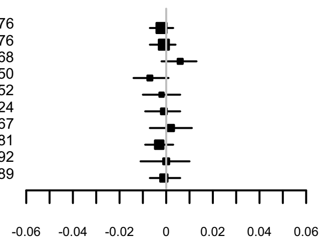

LGF Genetic Risk Scores in AMD Patients

Analysis	log(OR) [95% CI]	N
unadjusted	0.001 [-0.005 - 0.008]	33976
adjusted	0.001 [-0.006 - 0.008]	33976
<75 years	0.000 [-0.010 - 0.010]	16968
>75 years	0.004 [-0.006 - 0.014]	16650
male only	0.001 [-0.009 - 0.012]	14352
female only	0.001 [-0.008 - 0.010]	19624
GA	-0.002 [-0.014 - 0.010]	21067
CNV	0.000 [-0.007 - 0.008]	28581
GA&CNV	0.013 [-0.001 - 0.027]	19992
early AMD	0.002 [-0.007 - 0.011]	24489

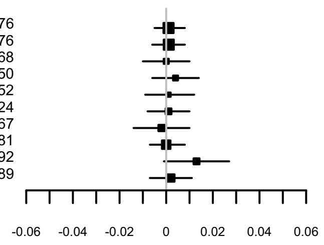

FVC Genetic Risk Scores in AMD Patients

Analysis	log(OR) [95% CI]	N
unadjusted	0.001 [-0.014 - 0.015]	33976
adjusted	0.000 [-0.015 - 0.016]	33976
<75 years	0.000 [-0.022 - 0.022]	16968
>75 years	0.003 [-0.019 - 0.025]	16650
male only	-0.002 [-0.025 - 0.021]	14352
female only	0.003 [-0.018 - 0.023]	19624
GA	0.011 [-0.015 - 0.038]	21067
CNV	-0.005 [-0.022 - 0.012]	28581
GA&CNV	0.009 [-0.022 - 0.041]	19992
early AMD	0.010 [-0.010 - 0.029]	24489

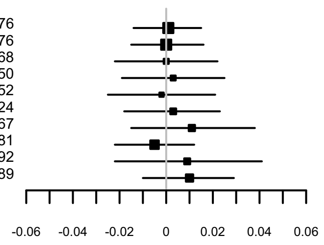

HTN Genetic Risk Scores in AMD Patients

Analysis	log(OR) [95% CI]	N
unadjusted	-0.023 [-0.034 - -0.012]	33976
adjusted	-0.021 [-0.033 - -0.010]	33976
<75 years	-0.023 [-0.040 - -0.006]	16968
>75 years	-0.023 [-0.040 - -0.006]	16650
male only	-0.026 [-0.043 - -0.008]	14352
female only	-0.018 [-0.034 - -0.003]	19624
GA	-0.028 [-0.048 - -0.008]	21067
CNV	-0.020 [-0.033 - -0.007]	28581
GA&CNV	-0.019 [-0.043 - 0.005]	19992
early AMD	0.000 [-0.015 - 0.015]	24489

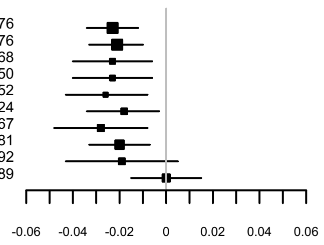

KRF Genetic Risk Scores in AMD Patients

Analysis	log(OR) [95% CI]	N
unadjusted	0.002 [-0.007 - 0.010]	33976
adjusted	0.002 [-0.007 - 0.012]	33976
<75 years	0.004 [-0.009 - 0.018]	16968
>75 years	0.000 [-0.014 - 0.013]	16650
male only	0.007 [-0.007 - 0.022]	14352
female only	-0.002 [-0.014 - 0.011]	19624
GA	0.005 [-0.011 - 0.021]	21067
CNV	0.003 [-0.007 - 0.014]	28581
GA&CNV	-0.008 [-0.027 - 0.011]	19992
early AMD	-0.008 [-0.020 - 0.004]	24489

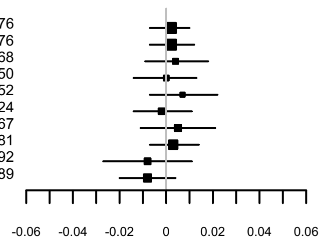

FEV1/FVC Genetic Risk Scores in AMD Patients

Analysis	log(OR) [95% CI]	N
unadjusted	0.006 [-0.002 - 0.014]	33976
adjusted	0.008 [-0.001 - 0.016]	33976
<75 years	0.006 [-0.006 - 0.018]	16968
>75 years	0.011 [-0.001 - 0.023]	16650
male only	0.010 [-0.003 - 0.023]	14352
female only	0.006 [-0.005 - 0.017]	19624
GA	0.000 [-0.014 - 0.015]	21067
CNV	0.010 [0.000 - 0.019]	28581
GA&CNV	0.011 [-0.006 - 0.028]	19992
early AMD	0.004 [-0.007 - 0.014]	24489

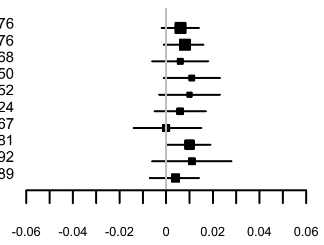

BMDFN Genetic Risk Scores in AMD Patients

Analysis	log(OR) [95% CI]	N
unadjusted	-0.007 [-0.012 - -0.003]	33976
adjusted	-0.009 [-0.013 - -0.004]	33976
<75 years	-0.008 [-0.015 - -0.001]	16968
>75 years	-0.009 [-0.016 - -0.001]	16650
male only	-0.006 [-0.014 - 0.001]	14352
female only	-0.010 [-0.017 - -0.004]	19624
GA	-0.008 [-0.017 - 0.000]	21067
CNV	-0.011 [-0.016 - -0.005]	28581
GA&CNV	0.000 [-0.010 - 0.010]	19992
early AMD	-0.003 [-0.009 - 0.003]	24489

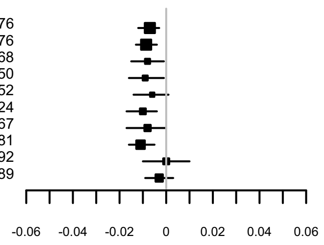

SBP Genetic Risk Scores in AMD Patients

Analysis	log(OR) [95% CI]	N
unadjusted	-0.013 [-0.020 - -0.005]	33976
adjusted	-0.011 [-0.019 - -0.003]	33976
<75 years	-0.014 [-0.025 - -0.003]	16968
>75 years	-0.009 [-0.020 - 0.002]	16650
male only	-0.011 [-0.023 - 0.001]	14352
female only	-0.011 [-0.022 - -0.001]	19624
GA	-0.016 [-0.029 - -0.002]	21067
CNV	-0.010 [-0.019 - -0.002]	28581
GA&CNV	-0.008 [-0.023 - 0.008]	19992
early AMD	0.007 [-0.003 - 0.016]	24489

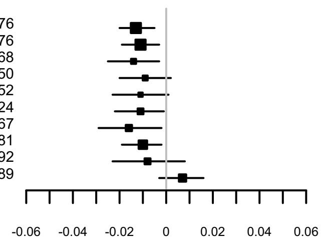

CREA Genetic Risk Scores in AMD Patients

Analysis	log(OR) [95% CI]	N
unadjusted	0.000 [-0.015 - 0.015]	33976
adjusted	0.002 [-0.014 - 0.017]	33976
<75 years	0.031 [0.009 - 0.054]	16968
>75 years	-0.023 [-0.046 - -0.001]	16650
male only	-0.023 [-0.047 - 0.001]	14352
female only	0.019 [-0.001 - 0.040]	19624
GA	0.008 [-0.018 - 0.035]	21067
CNV	-0.001 [-0.019 - 0.016]	28581
GA&CNV	-0.005 [-0.037 - 0.027]	19992
early AMD	0.033 [0.013 - 0.053]	24489

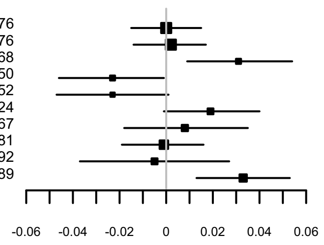

FEV1 Genetic Risk Scores in AMD Patients

Analysis	log(OR) [95% CI]	N
unadjusted	-0.006 [-0.020 - 0.008]	33976
adjusted	-0.011 [-0.025 - 0.004]	33976
<75 years	-0.012 [-0.033 - 0.010]	16968
>75 years	-0.008 [-0.030 - 0.013]	16650
male only	-0.008 [-0.031 - 0.014]	14352
female only	-0.012 [-0.032 - 0.008]	19624
GA	-0.015 [-0.040 - 0.010]	21067
CNV	-0.016 [-0.032 - 0.001]	28581
GA&CNV	0.026 [-0.004 - 0.055]	19992
early AMD	-0.017 [-0.036 - 0.002]	24489

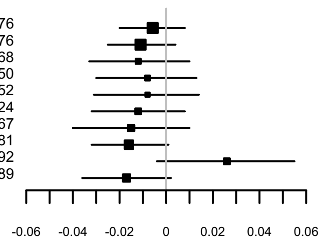

BMDLS Genetic Risk Scores in AMD Patients

Analysis	log(OR) [95% CI]	N
unadjusted	-0.006 [-0.011 - -0.002]	33976
adjusted	-0.006 [-0.011 - -0.001]	33976
<75 years	-0.008 [-0.015 - 0.000]	16968
>75 years	-0.005 [-0.012 - 0.003]	16650
male only	-0.007 [-0.014 - 0.001]	14352
female only	-0.005 [-0.012 - 0.001]	19624
GA	-0.007 [-0.016 - 0.001]	21067
CNV	-0.008 [-0.013 - -0.002]	28581
GA&CNV	0.004 [-0.006 - 0.015]	19992
early AMD	0.000 [-0.007 - 0.006]	24489

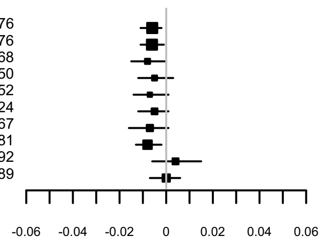

Supplementary Figure S2. continued

LEP Genetic Risk Scores in AMD Patients

Analysis	log(OR) [95% CI]	N
unadjusted	-0.002 [-0.006 - 0.003]	33976
adjusted	-0.002 [-0.007 - 0.003]	33976
<75 years	-0.004 [-0.011 - 0.004]	16968
>75 years	-0.002 [-0.009 - 0.005]	16650
male only	-0.006 [-0.014 - 0.001]	14352
female only	0.001 [-0.006 - 0.007]	19624
GA	-0.003 [-0.012 - 0.005]	21067
CNV	-0.002 [-0.008 - 0.004]	28581
GA&CNV	-0.003 [-0.013 - 0.008]	19992
early AMD	0.000 [-0.007 - 0.006]	24489

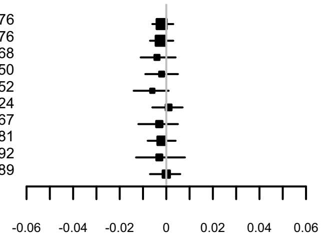

GGT Genetic Risk Scores in AMD Patients

Analysis	log(OR) [95% CI]	N
unadjusted	0.002 [-0.004 - 0.008]	33976
adjusted	0.001 [-0.005 - 0.008]	33976
<75 years	0.001 [-0.008 - 0.011]	16968
>75 years	0.000 [-0.009 - 0.010]	16650
male only	-0.003 [-0.013 - 0.007]	14352
female only	0.004 [-0.004 - 0.013]	19624
GA	0.005 [-0.007 - 0.016]	21067
CNV	0.000 [-0.007 - 0.007]	28581
GA&CNV	0.000 [-0.013 - 0.014]	19992
early AMD	0.004 [-0.004 - 0.013]	24489

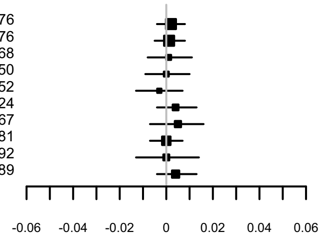

TC Genetic Risk Scores in AMD Patients

Analysis	log(OR) [95% CI]	N
unadjusted	-0.004 [-0.008 - -0.001]	33976
adjusted	-0.004 [-0.008 - 0.000]	33976
<75 years	-0.003 [-0.009 - 0.003]	16968
>75 years	-0.005 [-0.011 - 0.001]	16650
male only	-0.005 [-0.012 - 0.001]	14352
female only	-0.003 [-0.009 - 0.002]	19624
GA	-0.012 [-0.019 - -0.005]	21067
CNV	-0.002 [-0.006 - 0.003]	28581
GA&CNV	-0.006 [-0.014 - 0.003]	19992
early AMD	-0.004 [-0.009 - 0.001]	24489

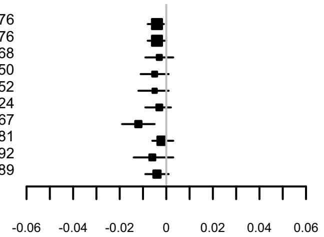

SUC Genetic Risk Scores in AMD Patients

Analysis	log(OR) [95% CI]	N
unadjusted	-0.001 [-0.005 - 0.004]	33976
adjusted	0.000 [-0.005 - 0.004]	33976
<75 years	-0.001 [-0.008 - 0.006]	16968
>75 years	0.000 [-0.007 - 0.007]	16650
male only	0.001 [-0.006 - 0.008]	14352
female only	-0.002 [-0.008 - 0.005]	19624
GA	0.005 [-0.003 - 0.013]	21067
CNV	-0.002 [-0.007 - 0.004]	28581
GA&CNV	0.000 [-0.010 - 0.009]	19992
early AMD	-0.003 [-0.009 - 0.003]	24489

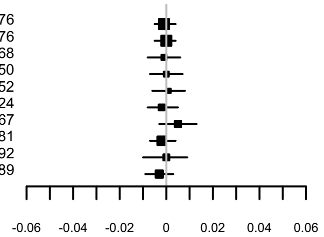

ALP Genetic Risk Scores in AMD Patients

Analysis	log(OR) [95% CI]	N
unadjusted	-0.010 [-0.018 - -0.002]	33976
adjusted	-0.011 [-0.020 - -0.003]	33976
<75 years	-0.013 [-0.025 - -0.001]	16968
>75 years	-0.011 [-0.024 - 0.001]	16650
male only	-0.012 [-0.025 - 0.001]	14352
female only	-0.011 [-0.022 - 0.001]	19624
GA	-0.025 [-0.039 - -0.010]	21067
CNV	-0.007 [-0.017 - 0.003]	28581
GA&CNV	-0.010 [-0.027 - 0.008]	19992
early AMD	-0.012 [-0.023 - -0.001]	24489

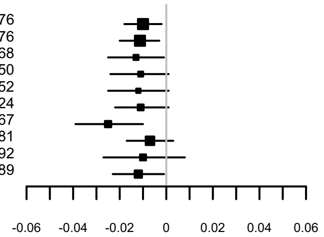

HDL Genetic Risk Scores in AMD Patients

Analysis	log(OR) [95% CI]	N
unadjusted	0.017 [0.013 - 0.020]	33976
adjusted	0.017 [0.014 - 0.021]	33976
<75 years	0.017 [0.012 - 0.022]	16968
>75 years	0.017 [0.012 - 0.023]	16650
male only	0.019 [0.013 - 0.025]	14352
female only	0.016 [0.011 - 0.021]	19624
GA	0.013 [0.006 - 0.019]	21067
CNV	0.018 [0.014 - 0.022]	28581
GA&CNV	0.022 [0.014 - 0.029]	19992
early AMD	0.018 [0.013 - 0.023]	24489

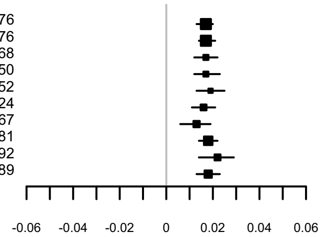

TG Genetic Risk Scores in AMD Patients

Analysis	log(OR) [95% CI]	N
unadjusted	-0.010 [-0.014 - -0.005]	33976
adjusted	-0.011 [-0.016 - -0.006]	33976
<75 years	-0.009 [-0.017 - -0.002]	16968
>75 years	-0.014 [-0.021 - -0.006]	16650
male only	-0.012 [-0.019 - -0.004]	14352
female only	-0.011 [-0.018 - -0.004]	19624
GA	-0.013 [-0.022 - -0.004]	21067
CNV	-0.010 [-0.016 - -0.004]	28581
GA&CNV	-0.013 [-0.024 - -0.003]	19992
early AMD	-0.015 [-0.022 - -0.008]	24489

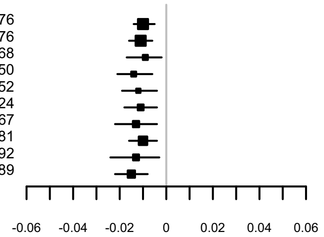

T2D Genetic Risk Scores in AMD Patients

Analysis	log(OR) [95% CI]	N
unadjusted	-0.005 [-0.010 - -0.001]	33976
adjusted	-0.005 [-0.010 - 0.000]	33976
<75 years	-0.006 [-0.013 - 0.001]	16968
>75 years	-0.004 [-0.010 - 0.003]	16650
male only	-0.004 [-0.011 - 0.003]	14352
female only	-0.005 [-0.012 - 0.001]	19624
GA	-0.002 [-0.010 - 0.006]	21067
CNV	-0.005 [-0.011 - 0.000]	28581
GA&CNV	-0.005 [-0.015 - 0.005]	19992
early AMD	-0.008 [-0.014 - -0.002]	24489

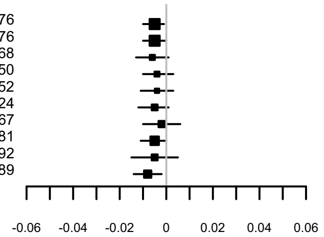

ALT Genetic Risk Scores in AMD Patients

Analysis	log(OR) [95% CI]	N
unadjusted	-0.010 [-0.025 - 0.006]	33976
adjusted	-0.006 [-0.022 - 0.010]	33976
<75 years	-0.015 [-0.038 - 0.009]	16968
>75 years	0.002 [-0.021 - 0.025]	16650
male only	-0.024 [-0.049 - 0.000]	14352
female only	0.007 [-0.014 - 0.029]	19624
GA	-0.018 [-0.045 - 0.010]	21067
CNV	0.001 [-0.018 - 0.019]	28581
GA&CNV	-0.011 [-0.044 - 0.022]	19992
early AMD	-0.008 [-0.029 - 0.012]	24489

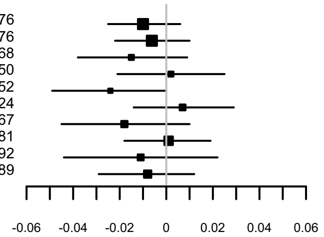

LDL Genetic Risk Scores in AMD Patients

Analysis	log(OR) [95% CI]	N
unadjusted	-0.013 [-0.017 - -0.009]	33976
adjusted	-0.013 [-0.017 - -0.008]	33976
<75 years	-0.011 [-0.018 - -0.005]	16968
>75 years	-0.014 [-0.021 - -0.008]	16650
male only	-0.013 [-0.019 - -0.006]	14352
female only	-0.013 [-0.019 - -0.007]	19624
GA	-0.022 [-0.029 - -0.014]	21067
CNV	-0.009 [-0.014 - -0.004]	28581
GA&CNV	-0.018 [-0.027 - -0.009]	19992
early AMD	-0.010 [-0.016 - -0.005]	24489

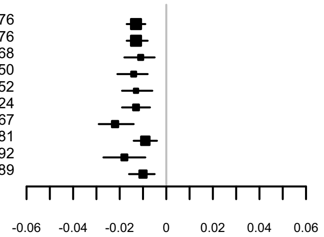

SCC Genetic Risk Scores in AMD Patients

Analysis	log(OR) [95% CI]	N
unadjusted	0.001 [-0.012 - 0.013]	33976
adjusted	-0.001 [-0.014 - 0.013]	33976
<75 years	0.009 [-0.011 - 0.028]	16968
>75 years	-0.013 [-0.033 - 0.006]	16650
male only	-0.004 [-0.025 - 0.017]	14352
female only	0.002 [-0.017 - 0.020]	19624
GA	-0.004 [-0.028 - 0.019]	21067
CNV	0.000 [-0.016 - 0.015]	28581
GA&CNV	0.004 [-0.023 - 0.032]	19992
early AMD	-0.007 [-0.024 - 0.011]	24489

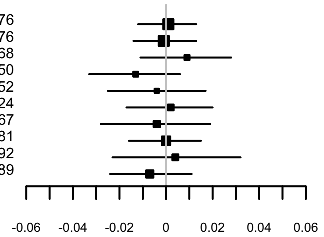

VitD Genetic Risk Scores in AMD Patients

Analysis	log(OR) [95% CI]	N
unadjusted	-0.003 [-0.020 - 0.015]	33976
adjusted	-0.004 [-0.022 - 0.014]	33976
<75 years	-0.004 [-0.030 - 0.023]	16968
>75 years	0.001 [-0.025 - 0.028]	16650
male only	0.009 [-0.019 - 0.037]	14352
female only	-0.014 [-0.038 - 0.011]	19624
GA	-0.032 [-0.063 - 0.000]	21067
CNV	0.005 [-0.015 - 0.026]	28581
GA&CNV	-0.008 [-0.046 - 0.030]	19992
early AMD	-0.005 [-0.028 - 0.018]	24489

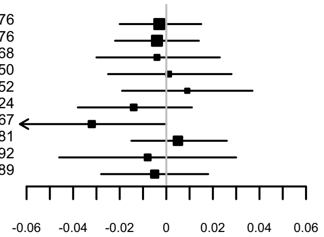

Supplementary Figure S2. continued

ALG Genetic Risk Scores in AMD Patients

Analysis	log(OR) [95% CI]	N
unadjusted	0.004 [-0.004 - 0.013]	33976
adjusted	0.004 [-0.005 - 0.014]	33976
<75 years	0.005 [-0.008 - 0.018]	16968
>75 years	0.004 [-0.009 - 0.017]	16650
male only	0.003 [-0.011 - 0.016]	14352
female only	0.006 [-0.006 - 0.018]	19624
GA	0.009 [-0.006 - 0.025]	21067
CNV	0.003 [-0.007 - 0.013]	28581
GA&CNV	0.007 [-0.012 - 0.026]	19992
early AMD	0.003 [-0.008 - 0.015]	24489

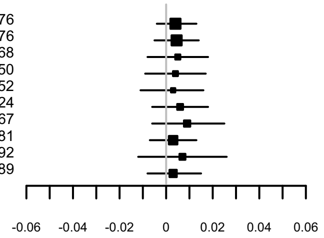

IBD Genetic Risk Scores in AMD Patients

Analysis	log(OR) [95% CI]	N
unadjusted	0.004 [0.001 - 0.007]	33976
adjusted	0.002 [-0.001 - 0.006]	33976
<75 years	0.002 [-0.002 - 0.007]	16968
>75 years	0.002 [-0.003 - 0.006]	16650
male only	0.003 [-0.002 - 0.008]	14352
female only	0.002 [-0.003 - 0.006]	19624
GA	0.003 [-0.003 - 0.008]	21067
CNV	0.003 [0.000 - 0.007]	28581
GA&CNV	0.000 [-0.007 - 0.006]	19992
early AMD	0.003 [-0.001 - 0.007]	24489

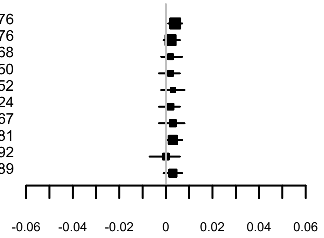

RA Genetic Risk Scores in AMD Patients

Analysis	log(OR) [95% CI]	N
unadjusted	0.005 [0.002 - 0.008]	33976
adjusted	0.006 [0.002 - 0.009]	33976
<75 years	0.004 [-0.001 - 0.009]	16968
>75 years	0.008 [0.003 - 0.013]	16650
male only	0.004 [-0.001 - 0.009]	14352
female only	0.008 [0.003 - 0.012]	19624
GA	0.011 [0.005 - 0.016]	21067
CNV	0.005 [0.001 - 0.009]	28581
GA&CNV	0.007 [0.000 - 0.015]	19992
early AMD	0.007 [0.003 - 0.011]	24489

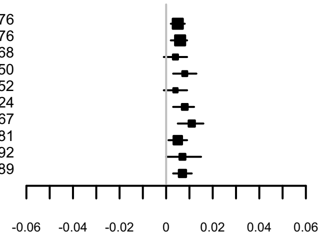

UC Genetic Risk Scores in AMD Patients

Analysis	log(OR) [95% CI]	N
unadjusted	0.005 [0.001 - 0.008]	33976
adjusted	0.003 [-0.001 - 0.007]	33976
<75 years	0.003 [-0.003 - 0.009]	16968
>75 years	0.002 [-0.003 - 0.008]	16650
male only	0.005 [-0.001 - 0.012]	14352
female only	0.000 [-0.005 - 0.006]	19624
GA	0.006 [0.000 - 0.013]	21067
CNV	0.002 [-0.002 - 0.007]	28581
GA&CNV	-0.001 [-0.009 - 0.007]	19992
early AMD	0.003 [-0.002 - 0.008]	24489

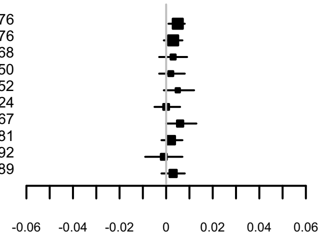

CD Genetic Risk Scores in AMD Patients

Analysis	log(OR) [95% CI]	N
unadjusted	0.003 [-0.001 - 0.006]	33976
adjusted	0.001 [-0.002 - 0.005]	33976
<75 years	0.002 [-0.003 - 0.007]	16968
>75 years	0.000 [-0.005 - 0.005]	16650
male only	0.004 [-0.001 - 0.009]	14352
female only	-0.001 [-0.005 - 0.004]	19624
GA	-0.001 [-0.006 - 0.005]	21067
CNV	0.003 [-0.001 - 0.007]	28581
GA&CNV	-0.001 [-0.008 - 0.006]	19992
early AMD	0.003 [-0.002 - 0.007]	24489

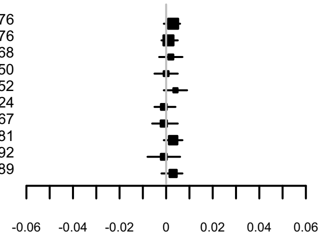

MS Genetic Risk Scores in AMD Patients

Analysis	log(OR) [95% CI]	N
unadjusted	0.003 [-0.002 - 0.008]	33976
adjusted	0.003 [-0.002 - 0.008]	33976
<75 years	0.005 [-0.003 - 0.012]	16968
>75 years	0.001 [-0.006 - 0.009]	16650
male only	0.009 [0.001 - 0.017]	14352
female only	-0.001 [-0.008 - 0.006]	19624
GA	0.000 [-0.009 - 0.008]	21067
CNV	0.002 [-0.004 - 0.008]	28581
GA&CNV	0.010 [0.000 - 0.021]	19992
early AMD	0.005 [-0.001 - 0.012]	24489

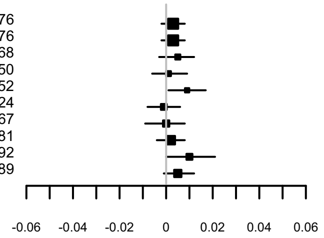

SLE Genetic Risk Scores in AMD Patients

Analysis	log(OR) [95% CI]	N
unadjusted	0.010 [0.004 - 0.015]	33976
adjusted	0.010 [0.004 - 0.016]	33976
<75 years	0.009 [0.000 - 0.018]	16968
>75 years	0.013 [0.004 - 0.022]	16650
male only	0.006 [-0.003 - 0.015]	14352
female only	0.013 [0.005 - 0.021]	19624
GA	0.012 [0.002 - 0.023]	21067
CNV	0.011 [0.004 - 0.018]	28581
GA&CNV	0.004 [-0.008 - 0.017]	19992
early AMD	0.009 [0.002 - 0.017]	24489

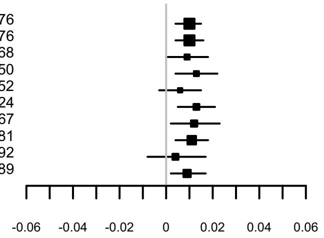

CRP Genetic Risk Scores in AMD Patients

Analysis	log(OR) [95% CI]	N
unadjusted	0.025 [0.018 - 0.032]	33976
adjusted	0.021 [0.013 - 0.029]	33976
<75 years	0.020 [0.009 - 0.031]	16968
>75 years	0.024 [0.013 - 0.035]	16650
male only	0.015 [0.003 - 0.027]	14352
female only	0.026 [0.016 - 0.036]	19624
GA	0.020 [0.006 - 0.033]	21067
CNV	0.019 [0.010 - 0.028]	28581
GA&CNV	0.038 [0.022 - 0.054]	19992
early AMD	0.016 [0.006 - 0.026]	24489

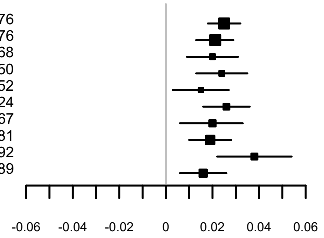

ECZ Genetic Risk Scores in AMD Patients

Analysis	log(OR) [95% CI]	N
unadjusted	-0.002 [-0.010 - 0.007]	33976
adjusted	0.003 [-0.006 - 0.013]	33976
<75 years	0.012 [-0.002 - 0.025]	16968
>75 years	-0.006 [-0.019 - 0.008]	16650
male only	0.006 [-0.008 - 0.021]	14352
female only	0.000 [-0.012 - 0.013]	19624
GA	0.009 [-0.007 - 0.025]	21067
CNV	-0.002 [-0.012 - 0.009]	28581
GA&CNV	0.007 [-0.012 - 0.027]	19992
early AMD	0.002 [-0.010 - 0.014]	24489

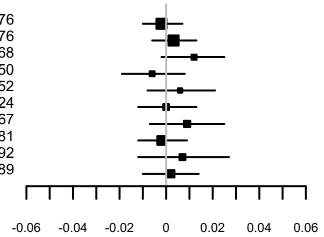

PSO Genetic Risk Scores in AMD Patients

Analysis	log(OR) [95% CI]	N
unadjusted	0.013 [0.008 - 0.019]	33976
adjusted	0.014 [0.009 - 0.020]	33976
<75 years	0.015 [0.007 - 0.023]	16968
>75 years	0.011 [0.003 - 0.019]	16650
male only	0.014 [0.005 - 0.022]	14352
female only	0.015 [0.007 - 0.022]	19624
GA	0.013 [0.004 - 0.023]	21067
CNV	0.013 [0.007 - 0.019]	28581
GA&CNV	0.019 [0.007 - 0.030]	19992
early AMD	0.013 [0.006 - 0.020]	24489

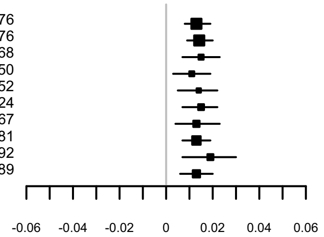

T1D Genetic Risk Scores in AMD Patients

Analysis	log(OR) [95% CI]	N
unadjusted	0.000 [-0.004 - 0.005]	33976
adjusted	0.001 [-0.003 - 0.006]	33976
<75 years	0.000 [-0.006 - 0.007]	16968
>75 years	0.002 [-0.005 - 0.009]	16650
male only	-0.002 [-0.009 - 0.005]	14352
female only	0.004 [-0.002 - 0.010]	19624
GA	0.001 [-0.007 - 0.009]	21067
CNV	0.000 [-0.005 - 0.005]	28581
GA&CNV	0.011 [0.001 - 0.020]	19992
early AMD	0.003 [-0.003 - 0.009]	24489

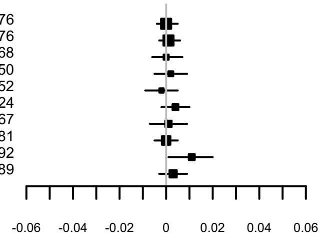

ICC Genetic Risk Scores in AMD Patients

Analysis	log(OR) [95% CI]	N
unadjusted	-0.003 [-0.005 - 0.000]	33976
adjusted	-0.002 [-0.005 - 0.000]	33976
<75 years	-0.002 [-0.005 - 0.002]	16968
>75 years	-0.003 [-0.006 - 0.001]	16650
male only	-0.004 [-0.007 - 0.000]	14352
female only	-0.001 [-0.004 - 0.002]	19624
GA	0.001 [-0.003 - 0.005]	21067
CNV	-0.004 [-0.007 - -0.001]	28581
GA&CNV	0.001 [-0.004 - 0.006]	19992
early AMD	0.000 [-0.003 - 0.003]	24489

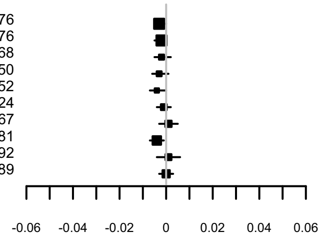

Supplementary Figure S2. continued

PICC Genetic Risk Scores in AMD Patients

Analysis	log(OR) [95% CI]	N
unadjusted	0.002 [-0.001 - 0.005]	33976
adjusted	0.002 [-0.001 - 0.004]	33976
<75 years	0.000 [-0.004 - 0.004]	16968
>75 years	0.003 [-0.001 - 0.007]	16650
male only	-0.001 [-0.005 - 0.004]	14352
female only	0.003 [0.000 - 0.007]	19624
GA	0.000 [-0.005 - 0.005]	21067
CNV	0.002 [-0.001 - 0.005]	28581
GA&CNV	0.003 [-0.003 - 0.008]	19992
early AMD	0.003 [-0.001 - 0.006]	24489

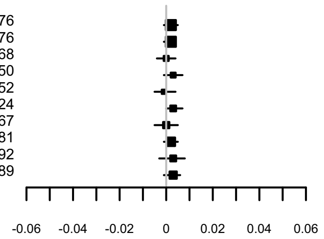

ERN Genetic Risk Scores in AMD Patients

Analysis	log(OR) [95% CI]	N
unadjusted	0.009 [0.000 - 0.017]	33976
adjusted	0.003 [-0.006 - 0.012]	33976
<75 years	0.014 [0.001 - 0.027]	16968
>75 years	-0.007 [-0.020 - 0.005]	16650
male only	0.005 [-0.009 - 0.018]	14352
female only	0.002 [-0.010 - 0.013]	19624
GA	-0.005 [-0.020 - 0.010]	21067
CNV	0.004 [-0.006 - 0.014]	28581
GA&CNV	-0.001 [-0.019 - 0.018]	19992
early AMD	-0.005 [-0.017 - 0.006]	24489

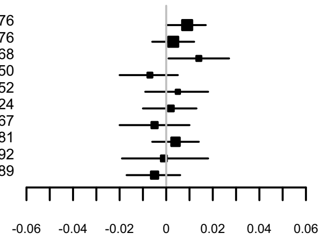

CMM Genetic Risk Scores in AMD Patients

Analysis	log(OR) [95% CI]	N
unadjusted	0.014 [0.005 - 0.022]	33976
adjusted	0.015 [0.006 - 0.024]	33976
<75 years	0.014 [0.001 - 0.027]	16968
>75 years	0.015 [0.002 - 0.028]	16650
male only	0.014 [0.000 - 0.028]	14352
female only	0.016 [0.004 - 0.028]	19624
GA	0.015 [-0.001 - 0.030]	21067
CNV	0.018 [0.008 - 0.028]	28581
GA&CNV	-0.001 [-0.019 - 0.017]	19992
early AMD	0.009 [-0.003 - 0.020]	24489

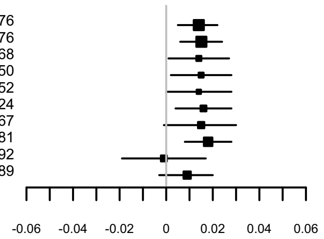

BMI Genetic Risk Scores in AMD Patients

Analysis	log(OR) [95% CI]	N
unadjusted	0.002 [-0.002 - 0.006]	33976
adjusted	0.005 [0.001 - 0.009]	33976
<75 years	0.006 [0.001 - 0.012]	16968
>75 years	0.003 [-0.003 - 0.009]	16650
male only	-0.001 [-0.007 - 0.005]	14352
female only	0.009 [0.004 - 0.014]	19624
GA	0.006 [-0.001 - 0.013]	21067
CNV	0.004 [0.000 - 0.009]	28581
GA&CNV	0.006 [-0.002 - 0.014]	19992
early AMD	0.000 [-0.005 - 0.005]	24489

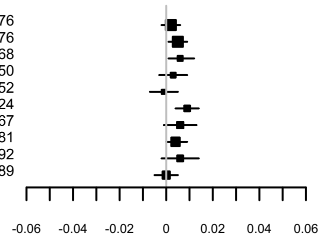

BRC Genetic Risk Scores in AMD Patients

Analysis	log(OR) [95% CI]	N
unadjusted	0.001 [-0.002 - 0.005]	33976
adjusted	0.000 [-0.003 - 0.004]	33976
<75 years	0.005 [0.000 - 0.010]	16968
>75 years	-0.005 [-0.010 - 0.001]	16650
male only	0.001 [-0.005 - 0.006]	14352
female only	0.000 [-0.005 - 0.004]	19624
GA	-0.001 [-0.007 - 0.005]	21067
CNV	-0.001 [-0.005 - 0.003]	28581
GA&CNV	0.003 [-0.005 - 0.010]	19992
early AMD	-0.001 [-0.006 - 0.003]	24489

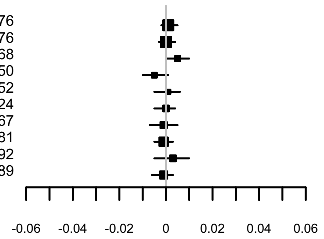

ERP Genetic Risk Scores in AMD Patients

Analysis	log(OR) [95% CI]	N
unadjusted	-0.002 [-0.007 - 0.003]	33976
adjusted	-0.002 [-0.007 - 0.003]	33976
<75 years	0.002 [-0.006 - 0.009]	16968
>75 years	-0.007 [-0.014 - 0.001]	16650
male only	-0.001 [-0.009 - 0.007]	14352
female only	-0.003 [-0.010 - 0.004]	19624
GA	-0.002 [-0.011 - 0.007]	21067
CNV	-0.004 [-0.010 - 0.002]	28581
GA&CNV	0.005 [-0.006 - 0.016]	19992
early AMD	0.000 [-0.006 - 0.007]	24489

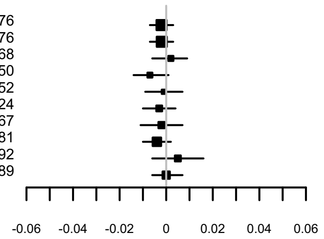

CRC Genetic Risk Scores in AMD Patients

Analysis	log(OR) [95% CI]	N
unadjusted	-0.003 [-0.012 - 0.005]	33976
adjusted	-0.001 [-0.010 - 0.008]	33976
<75 years	-0.005 [-0.018 - 0.007]	16968
>75 years	0.004 [-0.008 - 0.017]	16650
male only	-0.002 [-0.016 - 0.011]	14352
female only	0.000 [-0.011 - 0.012]	19624
GA	0.005 [-0.010 - 0.019]	21067
CNV	-0.002 [-0.012 - 0.008]	28581
GA&CNV	0.002 [-0.016 - 0.019]	19992
early AMD	0.005 [-0.006 - 0.016]	24489

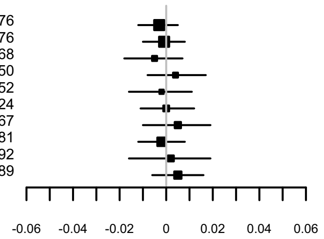

HGT Genetic Risk Scores in AMD Patients

Analysis	log(OR) [95% CI]	N
unadjusted	-0.001 [-0.004 - 0.001]	33976
adjusted	0.000 [-0.003 - 0.002]	33976
<75 years	-0.005 [-0.008 - -0.001]	16968
>75 years	0.004 [0.000 - 0.008]	16650
male only	-0.003 [-0.007 - 0.002]	14352
female only	0.001 [-0.002 - 0.005]	19624
GA	0.000 [-0.004 - 0.005]	21067
CNV	-0.001 [-0.004 - 0.002]	28581
GA&CNV	0.003 [-0.003 - 0.008]	19992
early AMD	0.001 [-0.002 - 0.005]	24489

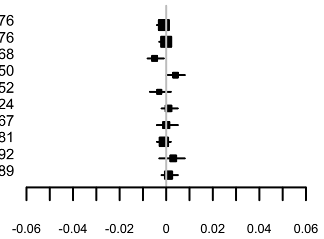

BRC only Genetic Risk Scores in AMD Patients

Analysis	log(OR) [95% CI]	N
unadjusted	0.001 [-0.003 - 0.005]	33976
adjusted	0.001 [-0.003 - 0.004]	33976
<75 years	0.004 [-0.001 - 0.010]	16968
>75 years	-0.003 [-0.009 - 0.002]	16650
male only	0.002 [-0.004 - 0.008]	14352
female only	0.000 [-0.006 - 0.005]	19624
GA	-0.001 [-0.007 - 0.006]	21067
CNV	0.000 [-0.004 - 0.004]	28581
GA&CNV	0.004 [-0.004 - 0.011]	19992
early AMD	0.000 [-0.005 - 0.004]	24489

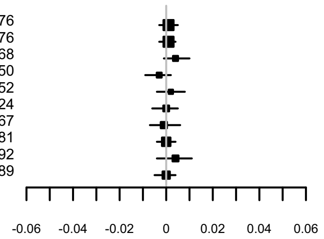

CLL Genetic Risk Scores in AMD Patients

Analysis	log(OR) [95% CI]	N
unadjusted	0.001 [-0.006 - 0.008]	33976
adjusted	0.001 [-0.006 - 0.008]	33976
<75 years	-0.004 [-0.014 - 0.007]	16968
>75 years	0.003 [-0.007 - 0.013]	16650
male only	-0.003 [-0.014 - 0.008]	14352
female only	0.003 [-0.006 - 0.013]	19624
GA	-0.003 [-0.015 - 0.009]	21067
CNV	0.002 [-0.006 - 0.010]	28581
GA&CNV	-0.003 [-0.018 - 0.012]	19992
early AMD	-0.005 [-0.014 - 0.004]	24489

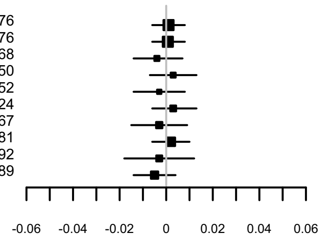

PRC Genetic Risk Scores in AMD Patients

Analysis	log(OR) [95% CI]	N
unadjusted	0.001 [-0.003 - 0.004]	33976
adjusted	0.002 [-0.002 - 0.006]	33976
<75 years	-0.001 [-0.006 - 0.005]	16968
>75 years	0.004 [-0.002 - 0.009]	16650
male only	0.003 [-0.003 - 0.009]	14352
female only	0.001 [-0.004 - 0.006]	19624
GA	-0.003 [-0.009 - 0.004]	21067
CNV	0.004 [-0.001 - 0.008]	28581
GA&CNV	0.002 [-0.006 - 0.010]	19992
early AMD	0.002 [-0.003 - 0.007]	24489

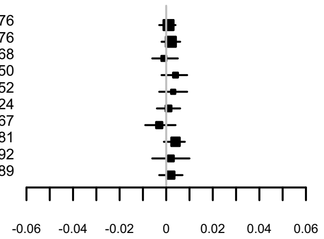

WHR Genetic Risk Scores in AMD Patients

Analysis	log(OR) [95% CI]	N
unadjusted	-0.003 [-0.008 - 0.002]	33976
adjusted	-0.003 [-0.008 - 0.003]	33976
<75 years	0.001 [-0.006 - 0.009]	16968
>75 years	-0.005 [-0.012 - 0.003]	16650
male only	0.000 [-0.008 - 0.008]	14352
female only	-0.004 [-0.011 - 0.002]	19624
GA	-0.003 [-0.012 - 0.006]	21067
CNV	-0.002 [-0.008 - 0.003]	28581
GA&CNV	-0.006 [-0.016 - 0.005]	19992
early AMD	-0.004 [-0.010 - 0.003]	24489

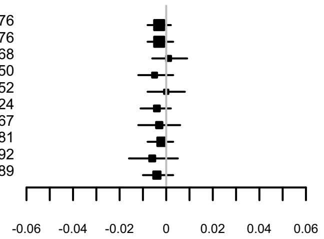

Supplement: Supplementary file 1 — Supplementary Figures S1 and S2 with Figure Legends. (PDF 1935 kb) [file 13073_2017_418_MOESM1_ESM.pdf]
